# Practice Variation in Perioperative Dexamethasone Use and Outcomes in Brain Metastasis Resection

**DOI:** 10.1001/jamanetworkopen.2025.4689

**Published:** 2025-04-11

**Authors:** David Wasilewski, Tommaso Araceli, Artem Rafaelian, Matthias Demetz, Benedikt Asey, Tunc-Faik Ersoy, Alice Dauth, Anne Neumeister, Ricarda Peukert, Paul Pöser, Christopher Krämer, Jan Bukatz, Zoe Shaked, Claudius Jelgersma, Anton Früh, Ran Xu, Martin Misch, David Capper, Felix Ehret, Nikolaj Frost, Lars Bullinger, Ulrich Keilholz, Christian Senft, Leon Schmidt, Harald Krenzlin, Florian Ringel, Anne Pohrt, Hanno S. Meyer, Jens Gempt, Johannes Kerschbaumer, Christian Freyschlag, Claudius Thomé, Matthias Simon, Daniel Dubinski, Thomas Freiman, Nils Ole Schmidt, Martin Proescholdt, Peter Vajkoczy, Julia Onken

**Affiliations:** 1 Department of Neurosurgery, Charité–Universitätsmedizin Berlin, corporate member of Freie Universität Berlin and Humboldt-Universität zu Berlin, Berlin, Germany; 2 Charité Comprehensive Cancer Center, Charité–Universitätsmedizin Berlin, corporate member of Freie Universität Berlin and Humboldt-Universität zu Berlin, Berlin, Germany; 3 German Cancer Consortium (DKTK), partner site Berlin, and German Cancer Research Center (DKFZ), Heidelberg, Germany; 4 Berlin Institute of Health at Charité–Universitätsmedizin Berlin, Berlin, Germany; 5 Department of Neurosurgery, University Regensburg Medical Center, Regensburg, Germany; 6 Wilhelm-Sander Neuro-Oncology Unit, University Regensburg Medical Center, Regensburg, Germany; 7 Department of Neurosurgery, University Medicine Rostock, Rostock, Germany; 8 Department of Neurosurgery, Medical University of Innsbruck, Innsbruck, Austria; 9 Department of Neurosurgery, University Medical Center Hamburg-Eppendorf, Hamburg, Germany; 10 Department of Neurosurgery (Evangelisches Klinikum Bethel), Medical School, Bielefeld University, Bielefeld, Germany; 11 Department of Neurosurgery, University Medical Center Mainz, Mainz, Germany; 12 Centre of Neuro-Oncology, Department of Neurosurgery, Jena University Hospital, Friedrich-Schiller-University Jena, Jena, Germany; 13 Institute of Neuropathology, Charité–Universitätsmedizin Berlin, corporate member of Freie Universität Berlin and Humboldt-Universität zu Berlin, Berlin, Germany; 14 Department of Radiation Oncology, Charité–Universitätsmedizin Berlin, corporate member of Freie Universität Berlin and Humboldt-Universität zu Berlin, Berlin, Germany; 15 Department of Infectious Diseases and Pulmonary Medicine, Charité–Universitätsmedizin Berlin, corporate member of Freie Universität Berlin and Humboldt-Universität zu Berlin, Berlin, Germany; 16 Department of Hematology, Oncology and Tumor Immunology, Charité–Universitätsmedizin Berlin, corporate member of Freie Universität Berlin and Humboldt-Universität zu Berlin, Berlin, Germany; 17 Institute of Biometry and Clinical Epidemiology, Charité–Universitätsmedizin Berlin, corporate member of Freie Universität Berlin and Humboldt-Universität zu Berlin, Berlin, Germany

## Abstract

**Importance:**

Variations in perioperative dexamethasone dosing are common in brain metastasis resection, but their impact on patient outcomes remains unclear.

**Objective:**

To evaluate the association between perioperative dexamethasone dosing and patient outcomes, focusing on overall survival (OS) and progression-free survival (PFS).

**Design, Setting, and Participants:**

This retrospective multicenter comparative effectiveness study used data collected from January 2010 to December 2023. Patients with symptomatic brain metastases undergoing primary surgical resection at 7 neurological centers in Germany and 1 in Austria and who had complete records of perioperative dexamethasone dosing were included. Propensity score matching (PSM) was used to control for confounders. Analysis was conducted from March to June 2024.

**Exposures:**

Cumulative perioperative dexamethasone administration over 27 days, dichotomized at 122 mg using maximally selected rank statistics.

**Main Outcomes and Measures:**

The primary outcome was OS. Secondary outcomes included extracranial PFS (ecPFS) and intracranial PFS (icPFS) as well as incidence of wound revision surgery after brain metastasis resection. Hazard ratios (HRs) were calculated using Cox proportional hazards models.

**Results:**

A total of 1064 patients were included in the analysis. The median (IQR) age was 64 (56-72) years, with 489 female patients (49%) and 541 male patients (51%). Non–small cell lung cancer (NSCLC) was the most common tumor entity (564 patients [53%]), followed by breast cancer (146 patients [14%]) and melanoma (138 patients [13%]). After PSM, patients receiving cumulative dexamethasone doses less than 122 mg had a median OS of 19.1 (95% CI, 15.2-22.4) months compared with 12.0 (95% CI, 9.1-14.7) months for those receiving 122 mg or more (*P* = .002). Multivariable analysis showed an independent association between higher cumulative dexamethasone doses and reduced OS (HR, 1.40; 95% CI, 1.18-1.66; *P* < .001). Secondary analyses demonstrated consistent findings with icPFS and ecPFS and a dose-response association between cumulative dexamethasone and hazard for death.

**Conclusions and Relevance:**

In this study, higher cumulative perioperative dexamethasone was associated with reduced OS, icPFS, and ecPFS in patients undergoing brain metastasis resection. These findings suggest that stricter dosing protocols could improve outcomes. Prospective trials are warranted to confirm these associations and guide evidence-based practice.

## Key Points

**Question:**

Is perioperative dexamethasone dosing associated with overall survival and progression-free survival in patients undergoing brain metastasis resection?

**Findings:**

In this comparative effectiveness study with 1064 patients, those exceeding the 122-mg cumulative dexamethasone cut point over 27 days had shorter overall survival than those receiving less than 122 mg cumulatively. The negative association was independent of clinical or prognostic factors, such as tumor volume, edema volume, disease-specific graded prognostic assessment, and adjuvant treatment class.

**Meaning:**

The findings of this study suggest that variations in perioperative dexamethasone dosing across centers may affect outcomes and stricter dosing protocols should be considered to minimize potential harm.

## Teaser

This comparative effectiveness study evaluates whether the cumulative dose of dexamethasone is associated with overall and progression-free survival among patients in Germany and Austria who underwent brain metastasis resection.

## Introduction

As many as 20% to 50% of patients with cancer develop brain metastasis during their disease.^1,2^ Symptomatic brain metastases often lead to neurological deficits due to mass effect of the metastases and associated perifocal edema. Depending on prognostic factors, such as clinical performance (Karnofsky Performance Status [KPS]), anatomical location, surgical accessibility, and symptom burden, patients typically undergo up-front neurosurgical resection followed by local adjuvant radiotherapy with or without systemic antitumor therapy.^3,4,5^ Advances in local therapies, such as stereotactic radiosurgery, and systemic treatments have significantly improved survival and intracranial control in these patients. Adjuvant therapies have shifted from postoperative radiotherapy and chemotherapy to postoperative radiotherapy with targeted therapies, including small molecule inhibitors and checkpoint inhibitors (CPIs).^3,4,5,6,7^

Similar to patients with glioblastoma, those with symptomatic brain metastases undergoing craniotomy and microsurgical brain resection regularly receive preoperative and postoperative dexamethasone to treat perifocal edema and alleviate neurological symptoms.^8,9^ However, dexamethasone use can be associated with significant adverse effects and immunosuppression.^10,11^ Retrospective case series and monocentric cohort studies from glioblastoma and brain metastases have been published, but larger studies on the adverse effects of dexamethasone and its association with postoperative treatments, including CPIs and targeted therapies, remain scarce. Systematic research is essential to optimize dosing and inform clinical decision-making.^12,13,14^

This multicenter retrospective study examines the association of cumulative perioperative dexamethasone doses (preoperative cumulative, postoperative cumulative, and total, ie, perioperative cumulative) with extracranial progression-free survival (ecPFS), intracranial PFS (icPFS), and overall survival (OS) as well as incidence of postoperative wound revision (POWR) surgery in 1064 patients undergoing brain metastasis resection. Practice variations in dosing were analyzed using descriptive statistics and propensity score matching (PSM) for covariate adjustment.

## Methods

### Patient Cohort and Data Collection

This multicenter retrospective comparative effectiveness study included patients who underwent primary brain metastasis resection between January 2010 and December 2023 at 7 neurosurgical university centers in Germany and 1 in Austria. Patient data were retrieved from institutional databases and manually reviewed. The study adhered to International Society for Pharmacoeconomics and Outcomes Research (ISPOR) and Strengthening the Reporting of Observational Studies in Epidemiology (STROBE) guidelines for study design and analysis. Exposure to perioperative dexamethasone, unless otherwise specified, was defined as the sum of cumulative preoperative (days −13 to −1) and postoperative (days 0 to 13) dexamethasone dosages in milligrams. Inclusion criteria required histologically confirmed brain metastases and complete perioperative dexamethasone documentation. Patients with uncertain prior dexamethasone use, incomplete dosage records, or corticosteroid use other than dexamethasone were excluded. Of 1780 screened patients, 1064 met the inclusion criteria.

### Ethical Review

This study complied with the Declaration of Helsinki.^15^ Written informed consent was obtained for neurosurgery and each center conducted the study approved by the local Ethics Committee, and data were collected under partners and the main site (Center 7) which provided institutional review board approval (EA1/140/24). The need for informed consent was waived for this study.

### Clinical Data and Patient Outcome Measures

Baseline was defined as the operation date of the first brain metastasis resection in each patient’s history. Patient data were retrieved from either paper records or electronic patient records and included the following baseline variables.

Clinical characteristics included variables such as age, sex, postoperative KPS, and disease-specific graded prognostic assessment (ds-GPA) at time of neurosurgical resection; ds-GPA scores were calculated according to the GPA index.^16,17^ Per definition, the ds-GPA encompasses information on tumor entity, age, presence of extracranial metastases, and intracranial metastasis burden as well as molecular pathology–related information.^16^ Further clinical variables included presence of underlying other diseases, defined as presence of cardiovascular diseases and chronic lung, kidney, or liver diseases. This was dichotomized as either not present or present.

Radiological characteristics included anatomical localization of the resected brain metastases, the brain region that was affected by brain metastases, the number of brain metastases at baseline, and the presence of extracranial metastases at baseline. These characteristics were based on reports from board-certified radiologists. Tumor volumes and associated edema volumes of the index metastasis, ie, resected brain metastasis, were quantified using a semiautomated 3-dimensional rendering algorithm in iPlannet (Brainlab) using the SmartBrush tool (T1-weighted images for tumor measurements and fluid-attenuated inversion recovery images for edema measurements). In case of multiple lesions, no addition of tumor or edema volumes was performed.

Additionally, all specimens underwent mandatory institutional pathological review by board-certified neuropathologists to determine histopathological characteristics. Data included tumor entity (World Health Organization classification), histopathological subtype, oncogenic driver mutations (eg, *EGFR*, *ALK*, *BRAF*), and programmed cell death ligand 1 tumor proportion score and immune cell score, where available. Treatment-related characteristics included primary tumor resection timing (preneurosurgery or postneurosurgery), local and/or systemic treatments prior to brain metastasis resection, and adjuvant therapies after resection, as previously described.

OS was calculated from the neurosurgical brain metastasis resection date to the last follow-up or death. Tumor progression was assessed using Response Evaluation Criteria in Solid Tumors version 1.1 and Response Assessment in Neuro-Oncology criteria based on computed tomography or cranial magnetic resonance imaging at the treating physician’s discretion. Progression was categorized as extracranial (ecPFS) or intracranial (icPFS). Median follow-up was determined using the reverse Kaplan-Meier method.

Another outcome parameter was the incidence of second surgical intervention, ie, POWR due to postoperative surgical site infection (SSI) after primary brain metastasis resection (index surgery) (date of operation until 6 months after index surgery). No further distinction between the type of wound infection was made.

### Statistical Analysis

Statistical analysis was performed using Prism version 9 (GraphPad Software) and R Studio version 2023.09.0 + 463 (R Project for Statistical Computing) to compute descriptive and inferential statistics as previously reported.^18^ The gtsummary package version 1.7.2 was used to describe tabular data of our patient cohort, including categorical and numerical variables. The χ^2^ test of independence was used to analyze the frequency table for categorical variables. Patient characteristics were described using medians and ranges for continuous variables and percentages for categorical variables. *t* Tests were 2-sided. A *P* value of .05 or less was considered statistically significant. We used the *t* test and the Mann-Whitney *U* test for group comparisons of continuous variables. Median OS was estimated by means of Kaplan-Meier analysis with confidence interval bands being displayed in the respective figures. Plotting was performed using the survival package version 3.5-8 and survminer package version 0.4.9. The association of each variable with the outcome was tested using the log-rank estimator. We tested the proportional hazards assumption with Schoenfeld residuals. Multivariable Cox regression was performed for matched data to assess the association of one or the simultaneous associations of multiple clinical variables with the outcome and was done using the survminer package.

We used the maximally selected rank statistic model by Lausen et al^19^ to identify optimal cut points for clinical outcomes. This approach examined the association between cumulative preoperative, postoperative, and total dexamethasone doses with OS, ecPFS, and icPFS. Cut points for cumulative perioperative dexamethasone doses were determined using the maximally selected rank statistics algorithm, implemented in the maxstat package version 0.7-25 and adjusted for covariate balancing through PSM.^19^ The association of perioperative dexamethasone dosing with survival was evaluated with a 1:1 ratio (optimal) PSM and a caliper set to 0.05, involving the following baseline covariates: tumor volume, edema volume, localization of brain metastases, ds-GPA, underlying diseases, postoperative adjuvant treatment, and underlying tumor entity, similar to our previous study.^18^ For PSM, we used the method of multiple imputation by chained equations (mice) from van Buuren and Groothuis-Oudshoorn^20^ and the mice package version 3.16.0 to impute missing variable values for tumor volume for 349 cases (32.80%), edema volume for 259 cases (24.34%), location for 7 cases (0.66%), ds-GPA for 155 cases (14.57%), and other diseases for 88 cases (8.27%). Other variables, such as underlying other diseases and extracranial metastases, had 8.46% (90 cases) and 1.79% (19 cases) missing data, respectively. The postoperative adjuvant therapies column had 9 missing cases (0.85%) (eFigure 1 in Supplement 1). No imputation was performed for this column. Further R packages used included common analytical packages such as dplyr version 1.1.4 and tidyverse version 2.0.0. Data collection was performed with Microsoft Excel version 14.3.9 (Microsoft Corp) and plotting with Prism or R studio.

## Results

### Patient Characteristics and Dexamethasone Dosage Practice Variation

The median (IQR) age of the 1064 patients in the cohort was 64 (56-72) years, with 489 (49%) female and 541 (51%) male patients. NSCLC was the most common tumor entity (564 [53%]), followed by breast cancer (146 [14%]) and melanoma (138 [13%]). The median follow-up time was 53.9 months (95% CI, 48.7-59.1 months) (eTable 1 in Supplement 1). Additional cohort characteristics are summarized in eTable 1 in Supplement 1. The mean dose of dexamethasone per center over the observation period is shown in eFigure 1 in Supplement 1. Median (IQR) cumulative dexamethasone doses were as follows: preoperatively, 48 (0-410) mg, postoperatively, 98 mg (0-354) mg, and perioperatively, 158 mg (0-668) mg (eTables 2-4 and eFigures 2-6 in Supplement 1). Significant differences in mean and median cumulative dexamethasone doses were observed among centers for preoperative, postoperative, and perioperative administration (eFigure 4 in Supplement 1). No significant correlations were found between cumulative dexamethasone doses (preoperative, postoperative, or perioperative) and tumor or edema volumes (eFigure 7 in Supplement 1).

### Patient Outcomes Before Matching and Cut Point Selection

**Figure 1.**
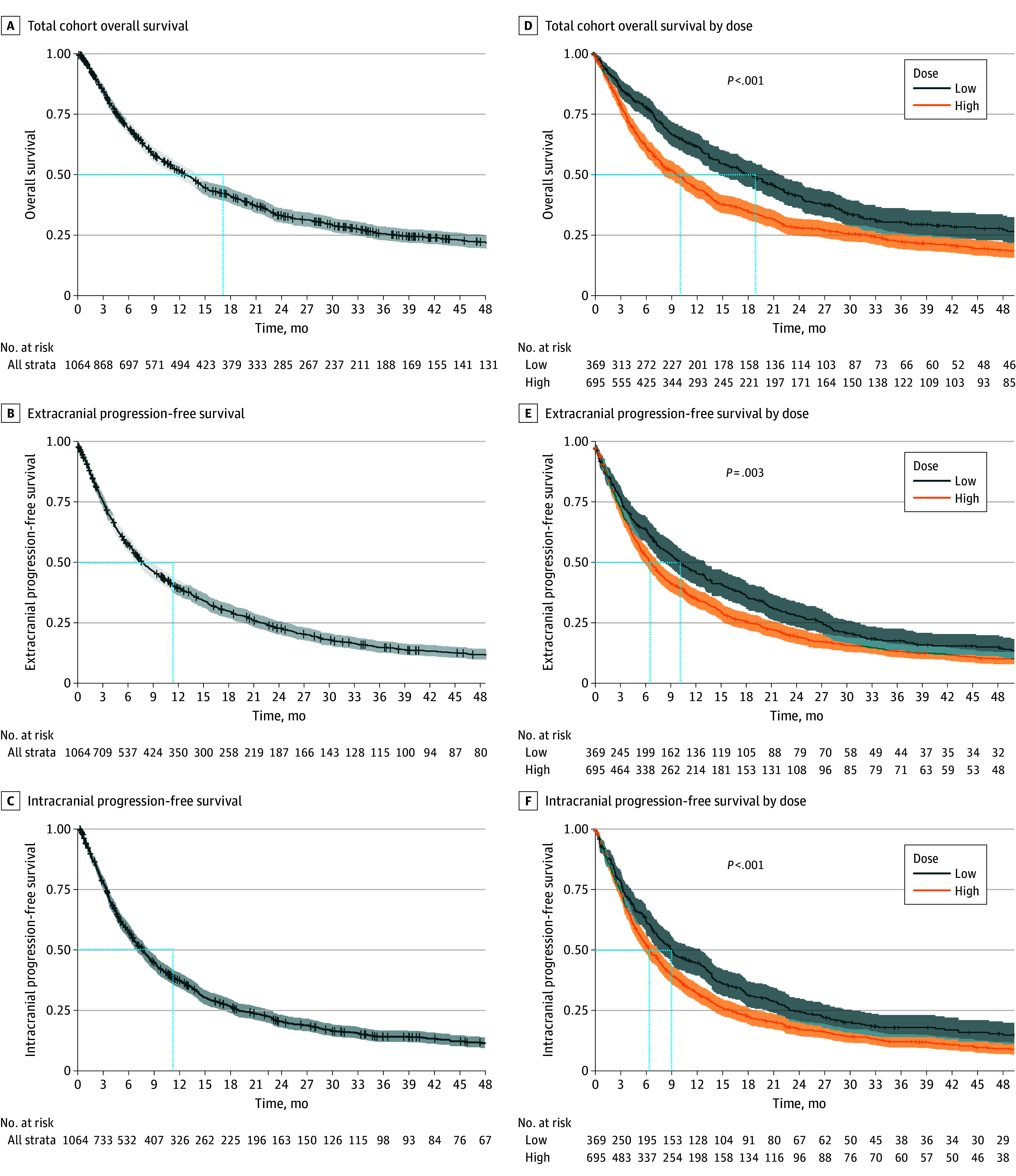
Kaplan-Meier Estimates for Patient Outcomes Overall and According to the Cut Point for Perioperative Dexamethasone Before Matching Kaplan-Meier curves and associated risk tables for the total cohort and median overall survival (A), extracranial progression-free survival (B), and intracranial progression-free survival (C). Kaplan-Meier curves and associated risk table displaying median overall survival (D), extracranial progression-free survival (E), and intracranial progression-free survival (F) before matching grouped into low (<122 mg) and high (≥122 mg) doses of dexamethasone. The cutoff of 122 mg was selected via maximally selected rank statistics (eFigure 9 in Supplement 1).

The median OS for the total cohort was 12.5 (95% CI, 11.1-14.0) months, with a median ecPFS of 7.5 (95% CI, 6.8-8.6) months and median icPFS of 7.4 (95% CI, 6.6-8.1) months (Figure 1A-C). The cut point for cumulative perioperative dexamethasone regarding OS was identified as 122 mg (*M* selected rank statistics = 4.6408, *P* < .001) (eTable 5 and eFigure 9 in Supplement 1). Before PSM, patients receiving less than 122 mg of dexamethasone perioperatively had a significantly longer median OS of 19.1 (95% CI, 15.2-22.4) months compared with 10.4 (95% CI, 8.5-11.8) months among those receiving 122 mg or greater (*P* < .001) (Figure 1D). Similarly, ecPFS and icPFS were significantly longer in the group that received less than 122 mg (ecPFS: 10.2 [95% CI, 8.2-13.2] months vs 6.6 [5.9-7.6] months; *P* = .003; icPFS: 9.2 [95% CI, 7.6-12.6] months vs 6.5 [95% CI, 5.8-7.6] months; *P* < .001) (Figure 1E-F).

Relevant covariates for PSM included tumor volume, edema volume, localization of brain metastases, ds-GPA, underlying diseases, postoperative adjuvant treatment, and underlying tumor entity. Significant differences between dexamethasone groups were observed for edema volume, ds-GPA, adjuvant therapy, and KPS, while no differences were noted for tumor entity, age, or sex (eTable 1 in Supplement 1).

Higher cumulative perioperative dexamethasone was significantly associated with POWR due to SSI (patients with POWR: mean dose, 204.8 mg; 95% CI, 175.2-234.3 mg; patients without POWR: mean dose, 176.5 mg; 95% CI, 168.9-184.1 mg). No significant associations were found for preoperative or postoperative dexamethasone doses (eTable 1 and eFigures 7 and 8 in Supplement 1).

### Patient Characteristics After Matching and Outcomes for Matched Data

**Figure 2.**
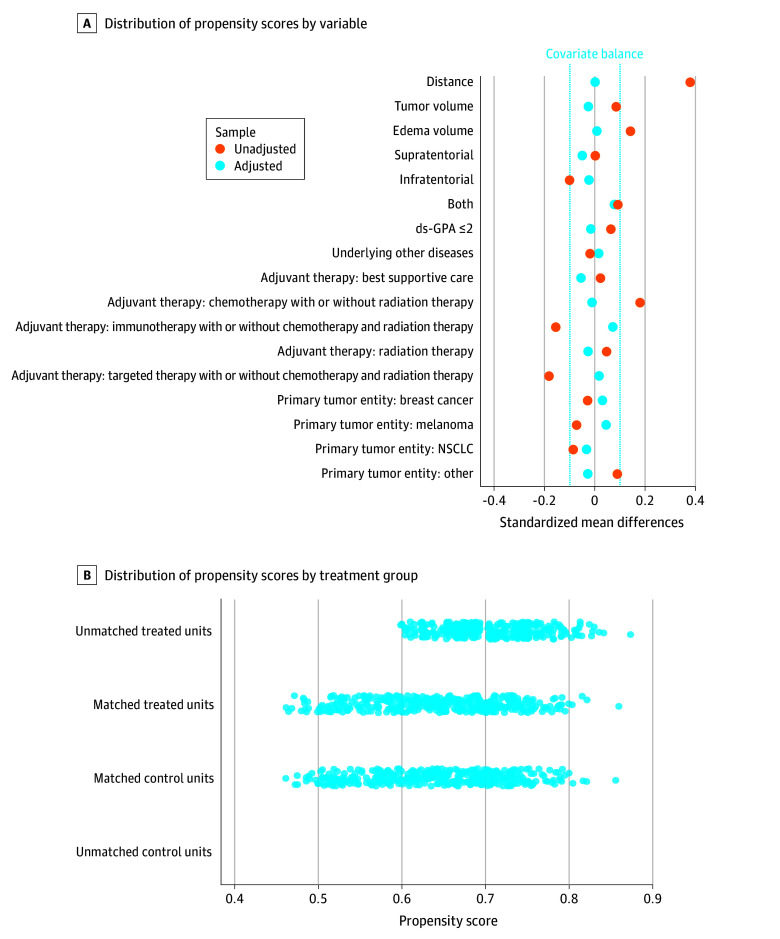
Distribution of Propensity Scores and Standardized Mean Differences for Matching Variables A loveplot displaying the standardized mean differences before (unadjusted in orange) and after propensity score matching (adjusted in blue) of each variable included into matching between treatment groups (A) and distribution of propensity scores (B). ds-GPA indicates disease-specific graded prognostic assessment; and NSCLC, non–small cell lung cancer.

**Figure 3.**
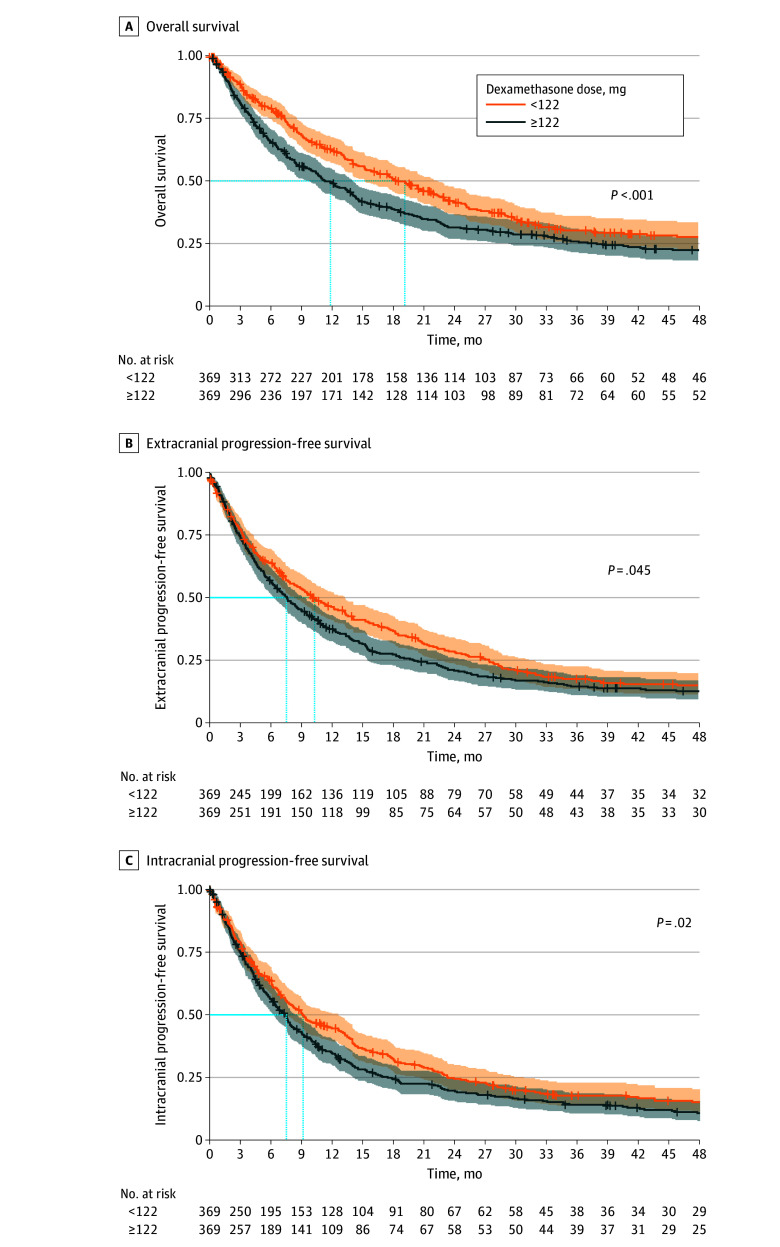
Kaplan-Meier Estimates for Patient Outcome by the Cut Point for Perioperative Dexamethasone After Matching Kaplan-Meier curves and associated risk table displaying median overall survival (A), extracranial progression-free survival (B), and intracranial progression-free survival (C) for the total cohort of patients after matching grouped into low (<122 mg) and high (≥122 mg) doses of dexamethasone. The cutoff of 122 mg was selected via maximally selected rank statistics (eFigure 9 in Supplement 1).

From the cohort of 1064 patients, 695 from the treatment group (≥122 mg dexamethasone) were matched with 369 from the control group (<122 mg dexamethasone) based on cumulative perioperative dexamethasone dosage. Postmatching analysis revealed no significant differences in tumor volume, edema volume, ds-GPA, comorbidities, or adjuvant therapy (Table and Figure 2A). Standard mean differences are shown in the love plot (Figure 2A). In the matched cohort, patients receiving less than 122 mg of dexamethasone had a median OS of 19.1 (95% CI, 15.2-22.4) months compared with 11.8 (95% CI, 9.87-14.1) months for those receiving 122 mg or more (*P* = .002). ecPFS was 10.2 (95% CI, 8.2-13.2) months vs 7.5 (95% CI, 6.3-9.3) months (*P* = .045), and icPFS was 9.2 (95% CI, 7.6-12.6) months vs 7.5 (95% CI, 6.3-8.6) months (*P* = .03) (Figure 3A-C).

**Table.** Comparison of the 2 Patient Groups After Propensity Score Matching

Variable	Patients, No. (%)			*P* value^b^	*q* Value^c^
	Overall (N = 738)	Treatment^a^			
		Less than cut point (n = 369)	Equal to or greater than cut point (n = 369)		
Tumor volume, median (IQR), mL	12 (6-22)	11 (6-22)	12 (6-22)	.69	>.99
Edema volume, median (IQR), mL	52 (24-95)	50 (23-91)	54 (25-96)	.42	>.99
ds-GPA score					
≥2	483 (65)	243 (66)	240 (65)	.82	>.99
<2	255 (35)	126 (34)	129 (35)		
Presence of other nononcological diseases					
No	312 (42)	155 (42)	157 (43)	.88	>.99
Yes	426 (58)	214 (58)	212 (57)		
Adjuvant postoperative therapy					
Best supportive care	64 (9)	32 (9)	32 (9)	.91	>.99
Chemotherapy and radiation therapy	135 (18)	72 (20)	63 (17)		
Immunotherapy, chemotherapy, and radiation therapy	171 (23)	83 (22)	88 (24)		
Radiation therapy	261 (35)	127 (34)	134 (36)		
Targeted therapy, chemotherapy, and radiation therapy	107 (14)	55 (15)	52 (14)		
Entity					
Breast cancer	104 (14)	53 (14)	51 (14)	>.99	>.99
Melanoma	88 (12)	44 (12)	44 (12)		
NSCLC	411 (56)	206 (56)	205 (56)		
Other	135 (18)	66 (18)	69 (19)		

Abbreviations: ds-GPA, disease-specific graded prognostic assessment; NSCLC, non–small cell lung cancer.

^a^ The optimal cut point value of 122 mg for perioperative dexamethasone was used to dichotomize patients.

^b^ Wilcoxon rank sum test; Pearson χ^2^ test.

^c^ False discovery rate correction for multiple testing.

**Figure 4.**
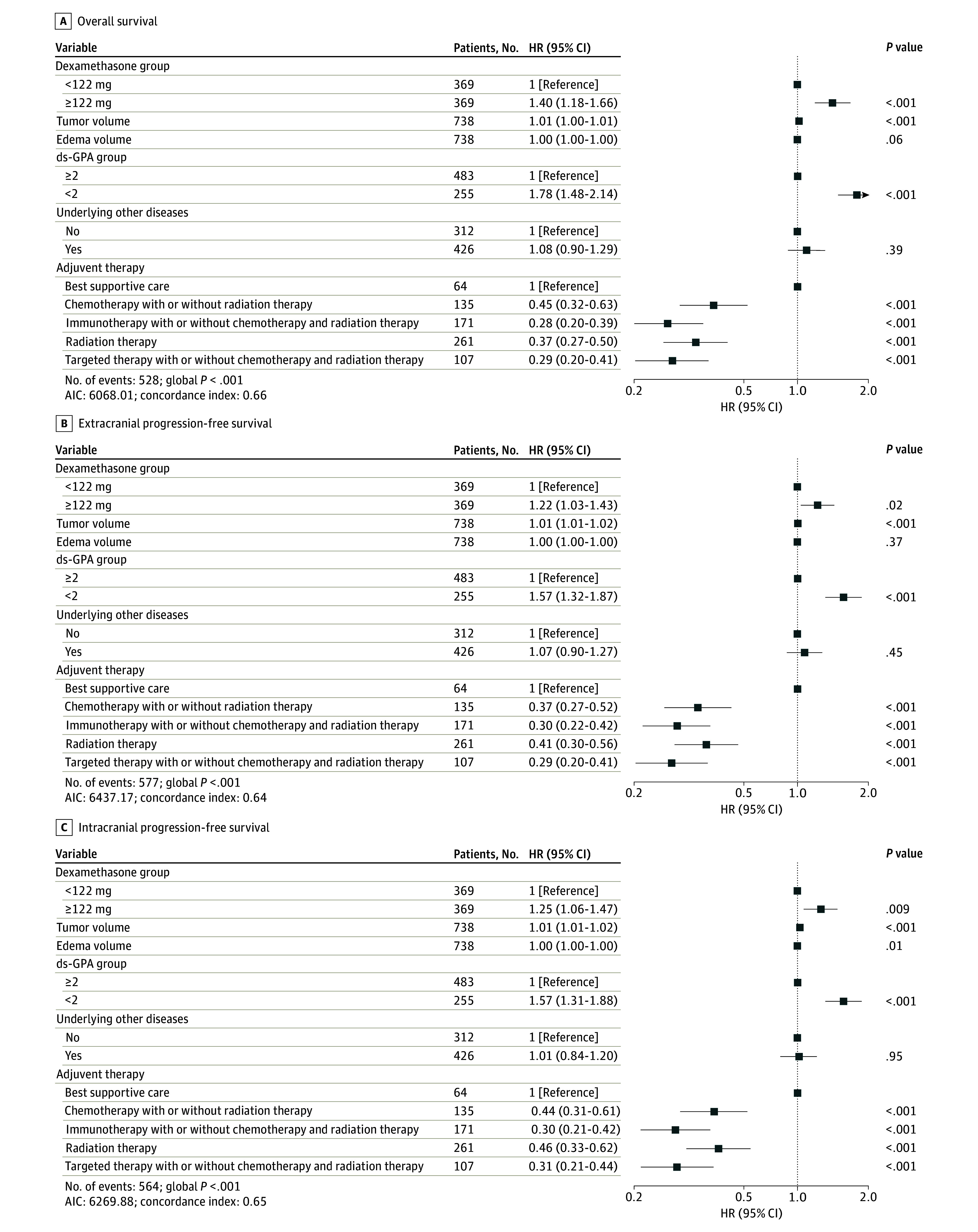
Multivariable Cox Regression for Patient Outcome After Matching Forest plots depict the hazard ratios and 95% CIs for overall survival (OS) (A), extracranial progression-free survival (ecPFS) (B), and intracranial PFS (icPFS) (C) for matched patient data; factors included into these models were dexamethasone dose, based on the 122-mg cut point; tumor volume; edema volume; disease-specific graded prognostic assessment (ds-GPA, dichotomized into GPA ≥2 and <2); presence of underlying other diseases; and type or class of adjuvant (postoperative) therapy.

In a Cox proportional hazards model, patients receiving 122 mg or more of dexamethasone had increased risk of death (HR, 1.40; 95% CI, 1.18-1.66; *P* < .001), as did those with a ds-GPA of less than 2 (HR, 1.78; 95% CI, 1.48-2.14; *P* < .001) (Figure 4A). The OS cut point was independently associated with ecPFS and icPFS, while ds-GPA less than 2 and postoperative treatment were significantly associated with all survival metrics (Figure 4A-C). Quartiles and spline regression demonstrated nonlinear associations between dexamethasone dose and mortality risk, with alternating intervals of increased and decreased hazard (eFigure 10 in Supplement 1).

Subgroup analysis for matched and unmatched data appear in eFigures 11 to 14 in Supplement 1. Analysis with unmatched data showed that the subgroup of patients with NSCLC displayed the largest difference at both cutoffs for cumulative dexamethasone (<122 mg: median OS, 21.7 [95% CI, 17.9-29.7] months; ≥122 mg: median OS, 9.2 [95% CI, 7.9-12.3] months; *P* < .001), while those with breast cancer, melanoma, and other cancers did not have significant differences. In a subgroup analysis, we analyzed the association of cumulative dexamethasone with outcomes among patients who received CPI as a postoperative systemic treatment. No association of cumulative dexamethasone with outcomes could be observed (data not shown).

## Discussion

The principal novel finding of the study is that cumulative perioperative dexamethasone dose of 122 mg or greater was independently associated with worse survival in patients with brain metastasis undergoing neurosurgical resection across 8 neurosurgical centers. Additionally, we observed a dose-dependent association between cumulative perioperative dexamethasone and OS.

Corticosteroids such as dexamethasone are a standard drug in the treatment of patients with brain metastases. They are used to control the mass effect of perifocal tumor edema and the resulting neurologic deficits.^12,21^ Treatment recommendations in this context lack evidence and are primarily based on expert opinion and few retrospective data.^3,4,10^ Remarkably, only 1 randomized study^22^ published in 1994 compared patients with brain metastases receiving either 4 mg, 8 mg, or 16 mg dexamethasone daily and reported improvement of symptoms by means of KPS at day 7 and day 28. There is significant variability between centers and treating physicians in dexamethasone initiation and tapering practices, as reflected in our data.^21,22,23^ To our knowledge, this retrospective multicenter comparative effectiveness study provides the first detailed analysis of daily dexamethasone dosing over an extended perioperative period and evaluates the association of cumulative preoperative, postoperative, and perioperative dexamethasone with patient outcomes. Consistent with prior survey studies, we observed substantial differences in preoperative and postoperative dexamethasone maintenance and tapering protocols across neurosurgical centers.^23^ Using cut point estimation, we identified optimal thresholds for survival estimation based on cumulative dexamethasone exposure. Notably, cumulative perioperative dexamethasone was associated with OS, while preoperative and postoperative dosing were not, likely due to the higher total exposure and more substantial biological effects associated with prolonged dexamethasone use. Our observations are in line with previously published research in the context of perioperative dexamethasone in patients with glioblastoma, although this study assessed a relatively long course of dexamethasone treatment not only postoperatively but essentially also preoperatively. For instance, Mistry et al^24^ found in a single-center PSM study that dexamethasone dosage on postoperative days 0 to 21 correlated with OS. Interestingly, Medikonda et al^25^ recently found that preoperative, but not postoperative or perioperative, dexamethasone dosage in patients with glioblastoma was associated with shorter OS and reported an HR of 3.0 (95% CI, 0.9-9.4), whereas the association of combined preoperative and postoperative dexamethasone with survival was less pronounced in our cohort.^25^ Interestingly, there was no significant correlation between cumulative dexamethasone dose and tumor volume or edema volume, which contrasts with the observation from Mistry and colleagues^24^ for glioblastoma. Our approach using the optimal PSM algorithm was associated with balancing the effect of important covariates that, on the one hand, are prognostic (eg, GPA score and postoperative [adjuvant] treatment) and, on the other hand, may represent confounding factors, such as edema volume and tumor volume but also tumor entity. Finally, the 122-mg cut point for cumulative perioperative dexamethasone was derived using a data-driven approach rather than predefined biological or clinical criteria. While dexamethasone is known to exhibit potent biological effects even at low doses, the increasing harm observed with higher doses likely reflects dose-dependent toxic effects, including immunosuppression.^12,13^ The sparsity of patients receiving higher doses introduces greater uncertainty in these estimates. This highlights the need for future studies to investigate clinically and biologically meaningful thresholds for dexamethasone dosing. Our subgroup analysis revealed a consistent negative association of cumulative dexamethasone doses of 122 mg or greater with survival across cancer types, although we observed a significant difference between dexamethasone groups only among patients with NSCLC. In contrast, in breast cancer and other cancers there was a less pronounced difference, and interestingly, there was an even weaker difference between dexamethasone groups in the case of melanoma. The preponderance of patients with NSCLC may disproportionately affect the results, while smaller sample sizes for melanoma and other subgroups limited statistical power. Despite this, the findings support the clinical relevance of dexamethasone dosing thresholds and highlight the need for validation in larger, more balanced cohorts.

### Limitations

This study has several limitations. While it includes a large dataset from multiple neurosurgical centers, the retrospective design poses inherent challenges. We note that detailed characteristics of excluded patients were unavailable, limiting any comprehensive bias assessment or direct comparison with included patients. However, our final cohort is representative of typical demographic characteristics (tumor types, age, sex) for brain metastasis studies, and thus likely generalizable within the context of existing literature.^6,7,8,22^

The reduced association of cumulative perioperative dexamethasone with ecPFS and icPFS after covariate balancing likely reflects inconsistent follow-up imaging. Preoperative dexamethasone records may not fully capture actual administration, especially in cases involving institutional transfers. Patients with incomplete dexamethasone documentation were excluded, potentially introducing selection bias.

Our infection definition focused on revision operations for SSI, excluding systemic infections such as fatal sepsis or cases where patients chose palliation over revision. The association between perioperative dexamethasone and SSIs may diminish over time, making operations performed beyond 6 months less likely to be causally linked to perioperative dexamethasone use. Broader infection definitions and time-dependent analyses should be explored in future studies. The long observation period also reflects evolving systemic treatment landscapes for patients with brain metastases, further complicating comparisons.

Prospective studies or randomized clinical trials focusing on patients with symptomatic brain metastasis are needed to evaluate the biological effects of dexamethasone, such as its influence on the systemic and local tumor immune microenvironment.^11,21,22,23,26,27,28,29^ Additionally, this study is limited to surgical patients, who are typically more symptomatic, excluding those treated nonsurgically. As CPI and kinase-targeting therapies gain prominence, dexamethasone dosing and tapering strategies will likely become even more critical. While previous studies suggest that corticosteroid timing affects CPI response, with earlier administration associated with poorer survival, our analysis did not observe significant survival differences among patients who underwent resection receiving postoperative CPIs.^30,31,32^ However, this may reflect the limited number of patients treated with postoperative CPIs. Further studies are needed to clarify dexamethasone’s role in modulating CPI efficacy after resection. Furthermore, this study did not explore the pleiotropic effects of dexamethasone on the central nervous system microenvironment or peripheral immune function, which warrants further investigation.^11,12^

## Conclusions

This comparative effectiveness study of patients with resected brain metastases across 8 neurosurgical centers highlights the association of cumulative perioperative dexamethasone with OS, PFS, and SSI requiring wound revision. Future research should focus on refining dexamethasone dosing strategies and exploring its impact on specific patient subgroups and biological outcomes.

## Notes

Supplement 1. **eFigure 1.** Plotting Missing Values Before Data Imputation **eFigure 2.** Comparison of Mean Dexamethasone Dosing Over Longitudinally Study Period of 27 Days **eFigure 3.** Comparative Analysis of Cumulative Dexamethasone Administration **eFigure 4.** Comparative Analysis of Cumulative Dexamethasone Administration Across Centers **eFigure 5.** Temporal Distribution of Perioperative Dexamethasone Dosage Across Treatment Centers **eFigure 6.** Time Course of Mean Perioperative Dexamethasone per Center **eFigure 7.** Correlations Between Numerical Clinical Covariates **eFigure 8.** Association Between Cumulative Dexamethasone and Postoperative Wound Revision Surgery **eFigure 9.** Cut Point Selection Using Maximally Selected Rank Statistics **eFigure 10.** Quartiles and Spline Regression to Model Dose-Dependency of Cumulative Perioperative Dexamethasone on the Hazard of Death **eFigure 11.** Tumor-Specific Subgroup KM Analysis for Dexamethasone 122-mg Cutoff—Unmatched Data **eFigure 12.** Tumor-Specific Subgroup Cox Proportional Hazard Regression Analysis for Dexamethasone 122-mg Cutoff—Unmatched Data **eFigure 13.** Tumor-specific Subgroup KM Analysis for Dexamethasone 122-mg Cutoff—Matched Data **eFigure 14.** Tumor-Specific Subgroup Cox Proportional Hazard Regression Analysis for Dexamethasone 122-mg Cutoff—Matched Data **eTable 1.** Patient Characteristics of the Total Cohort Before Matching **eTable 2.** Statistical Summary of Perioperative Dexamethasone Doses Across Different Centers **eTable 3.** Statistical Summary of Preoperative Dexamethasone Doses Across Different Centers **eTable 4.** Statistical Summary of Postoperative Dexamethasone Doses Across Different Centers **eTable 5.** Summary of Cut Point Values of Perioperative, Preoperative, and Postoperative Dexamethasone for Patient Outcome Using Maximally Selected Rank Statistics

Supplement 2.
Data Sharing Statement
