# Supplementary material for: Practice Variation in Perioperative Dexamethasone Use and Outcomes in Brain Metastasis Resection
Source: JAMA Netw Open. 2025 Apr 11;8(4):e254689. doi: 10.1001/jamanetworkopen.2025.4689 (PMC11992604; doi:10.1001/jamanetworkopen.2025.4689)
Supplement: Supplement 1. — eFigure 1. Plotting Missing Values Before Data Imputation eFigure 2. Comparison of Mean Dexamethasone Dosing Over Longitudinally Study Period of 27 Days eFigure 3. Comparative Analysis of Cumulative Dexamethasone Administration eFigure 4. Comparative Analysis of Cumulative Dexamethasone Administration Across Centers eFigure 5. Temporal Distribution of Perioperative Dexamethasone Dosage Across Treatment Centers eFigure 6. Time Course of Mean Perioperative Dexamethasone per Center eFigure 7. Correlations Between Numerical Clinical Covariates eFigure 8. Association Between Cumulative Dexamethasone and Postoperative Wound Revision Surgery eFigure 9. Cut Point Selection Using Maximally Selected Rank Statistics eFigure 10. Quartiles and Spline Regression to Model Dose-Dependency of Cumulative Perioperative Dexamethasone on the Hazard of Death eFigure 11. Tumor-Specific Subgroup KM Analysis for Dexamethasone 122-mg Cutoff—Unmatched Data eFigure 12. Tumor-Specific Subgroup Cox Proportional Hazard Regression Analysis for Dexamethasone 122-mg Cutoff—Unmatched Data eFigure 13. Tumor-specific Subgroup KM Analysis for Dexamethasone 122-mg Cutoff—Matched Data eFigure 14. Tumor-Specific Subgroup Cox Proportional Hazard Regression Analysis for Dexamethasone 122-mg Cutoff—Matched Data eTable 1. Patient Characteristics of the Total Cohort Before Matching eTable 2. Statistical Summary of Perioperative Dexamethasone Doses Across Different Centers eTable 3. Statistical Summary of Preoperative Dexamethasone Doses Across Different Centers eTable 4. Statistical Summary of Postoperative Dexamethasone Doses Across Different Centers eTable 5. Summary of Cut Point Values of Perioperative, Preoperative, and Postoperative Dexamethasone for Patient Outcome Using Maximally Selected Rank Statistics [file jamanetwopen-e254689-s001.pdf]

Supplemental Online Content

Wasilewski D, Araceli T, Rafaelian A, et al. Practice variation in perioperative dexamethasone use and outcomes in brain metastasis resection. *JAMA Netw Open.* 2025;8(4):e254689. doi:10.1001/jamanetworkopen.2025.4689

eFigure 1. Plotting Missing Values Before Data Imputation

eFigure 2. Comparison of Mean Dexamethasone Dosing Over Longitudinally Study Period of 27 Days

eFigure 3. Comparative Analysis of Cumulative Dexamethasone Administration

eFigure 4. Comparative Analysis of Cumulative Dexamethasone Administration Across Centers

eFigure 5. Temporal Distribution of Perioperative Dexamethasone Dosage Across Treatment Centers

eFigure 6. Time Course of Mean Perioperative Dexamethasone per Center

eFigure 7. Correlations Between Numerical Clinical Covariates

eFigure 8. Association Between Cumulative Dexamethasone and Postoperative Wound Revision Surgery

eFigure 9. Cut Point Selection Using Maximally Selected Rank Statistics

eFigure 10. Quartiles and Spline Regression to Model Dose-Dependency of Cumulative Perioperative Dexamethasone on the Hazard of Death

eFigure 11. Tumor-Specific Subgroup KM Analysis for Dexamethasone 122-mg Cutoff—Unmatched Data

eFigure 12. Tumor-Specific Subgroup Cox Proportional Hazard Regression Analysis for Dexamethasone 122-mg Cutoff—Unmatched Data

eFigure 13. Tumor-specific Subgroup KM Analysis for Dexamethasone 122-mg Cutoff—Matched Data

eFigure 14. Tumor-Specific Subgroup Cox Proportional Hazard Regression Analysis for Dexamethasone 122-mg Cutoff—Matched Data

© 2025 Wasilewski D et al. *JAMA Network Open*.

eTable 1. Patient Characteristics of the Total Cohort Before Matching

eTable 2. Statistical Summary of Perioperative Dexamethasone Doses Across Different Centers

eTable 3. Statistical Summary of Preoperative Dexamethasone Doses Across Different Centers

eTable 4. Statistical Summary of Postoperative Dexamethasone Doses Across Different Centers

eTable 5. Summary of Cut Point Values of Perioperative, Preoperative, and Postoperative Dexamethasone for Patient Outcome Using Maximally Selected Rank Statistics

This supplemental material has been provided by the authors to give readers additional information about their work.

© 2025 Wasilewski D et al. *JAMA Network Open*.

eFigure 1. Plotting Missing Values Before Data Imputation

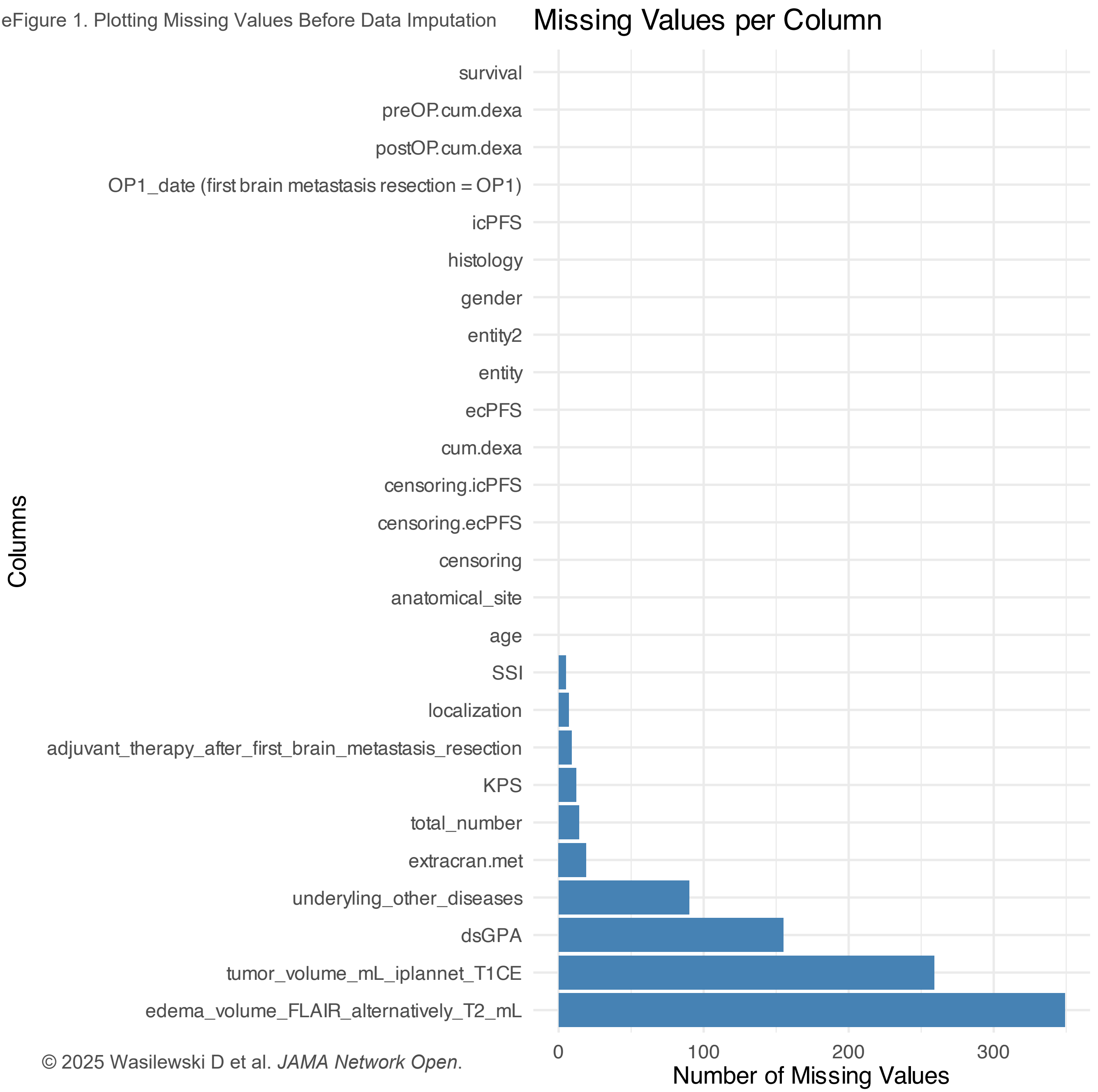

© 2025 Wasilewski D et al. *JAMA Network Open*.

The figure visualizes the number of missing values across selected columns in the dataset. The bar chart shows the absolute count of missing data for variables such as edema volume, tumor volume, ds-GPA, underlying other diseases, and other key clinical factors. Columns with the highest proportion of missing data include "edema volume" (32.80%) and "tumor volume" (24.34%), while other variables like "post-operative adjuvant therapies" (0.85%) and "location" (0.66%) have minimal missing values. This representation highlights the extent of missing data and informs the data imputation strategy applied during analysis.

© 2025 Wasilewski D et al. *JAMA Network Open*.

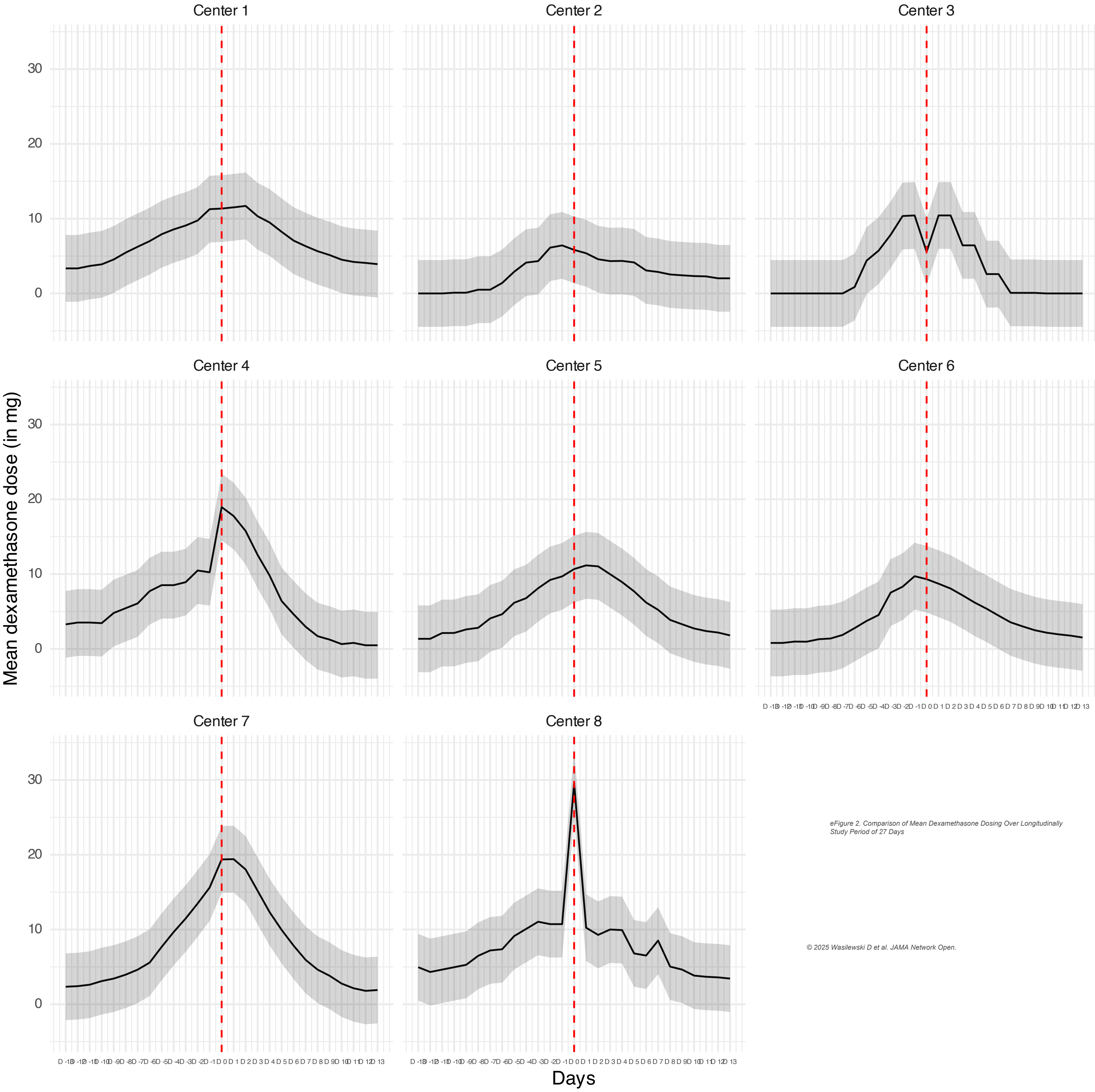

eFigure 2. Comparison of Mean Dexamethasone Dosing Over Longitudinally Study Period of 27 Days

© 2025 Wasilewski D et al. JAMA Network Open.

eFigure 2. Comparison of Mean Dexamethasone Dosing Over Longitudinally Study Period of 27 Days

This figure illustrates the daily mean dexamethasone dosage administered across all participating centers over the longitudinal study period. 95% CI is indicated with the gray overlay. Day of brain metastasis resection is marked with a vertical dotted red line. Variations in dosage trends between centers are highlighted to emphasize differences in practice patterns.

© 2025 Wasilewski D et al. *JAMA Network Open*.

eFigure 3. Comparative Analysis of Cumulative Dexamethasone Administration

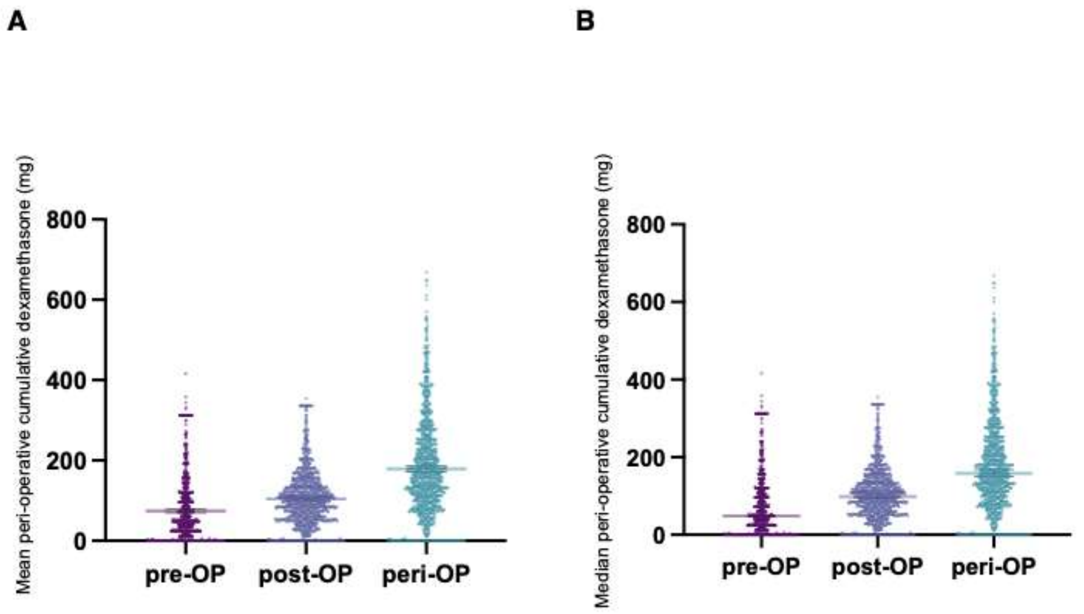

Mean peri-operative cumulative dexamethasone dose in mg administered between day -13 before operation and day 13 after operation in resected brain metastases (**A**) and median Mean peri-operative cumulative dexamethasone dose in mg administered between day -13 before operation and day 13 after operation in resected brain metastases (**B**). Data are shown for all centers.

© 2025 Wasilewski D et al. *JAMA Network Open*.

eFigure 4. Comparative Analysis of Cumulative Dexamethasone Administration Across Centers

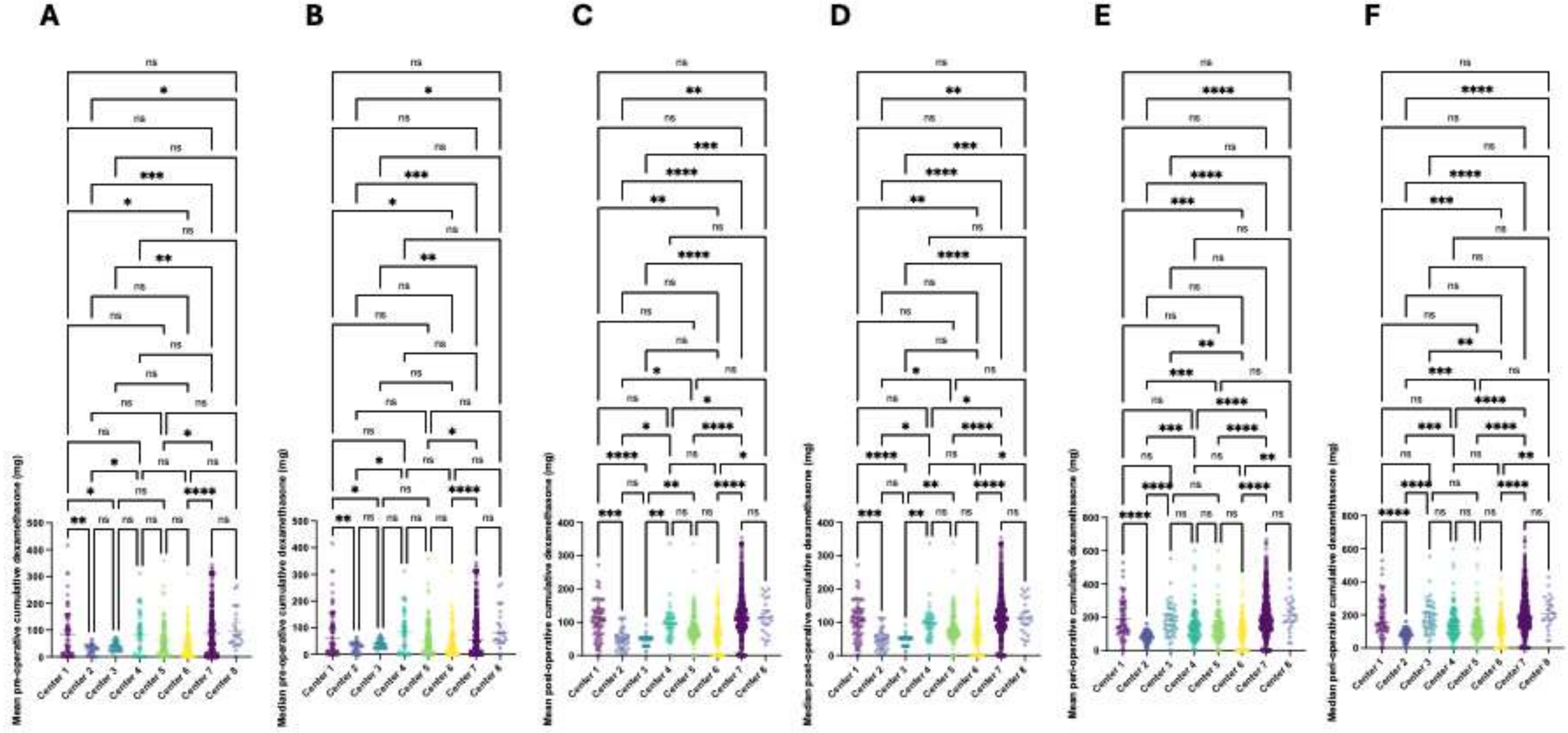

Mean peri-operative cumulative dexamethasone dose in mg administered between day -13 before operation and day -1 before operation in resected brain metastases for each center (**A**), and median pre-operative cumulative dexamethasone dose (**B**). Mean

© 2025 Wasilewski D et al. *JAMA Network Open*.

and median post-operative cumulative dexamethasone dose in mg administered per center between day 0 before operation and day 13 after operation in resected brain metastases (**C, D**). Mean and median peri-operative cumulative dexamethasone dose in mg administered per center between day -13 before operation and day 13 after operation in resected brain metastases (**E, F**). The ANOVA summary shows a significant F statistic of 23.40 with a p-value less than 0.0001, indicating that there are statistically significant differences in dexamethasone dosages among the centers. Multiple comparisons were reported using one-way ANOVA, Tukey's multiple comparisons test, where p values were usually considered statistically significant if <.05, and labeled as *P < .05, **P < .01, and ***P < .001.

© 2025 Wasilewski D et al. *JAMA Network Open*.

eFigure 5. Temporal Distribution of Perioperative Dexamethasone Dosage Across Treatment Centers

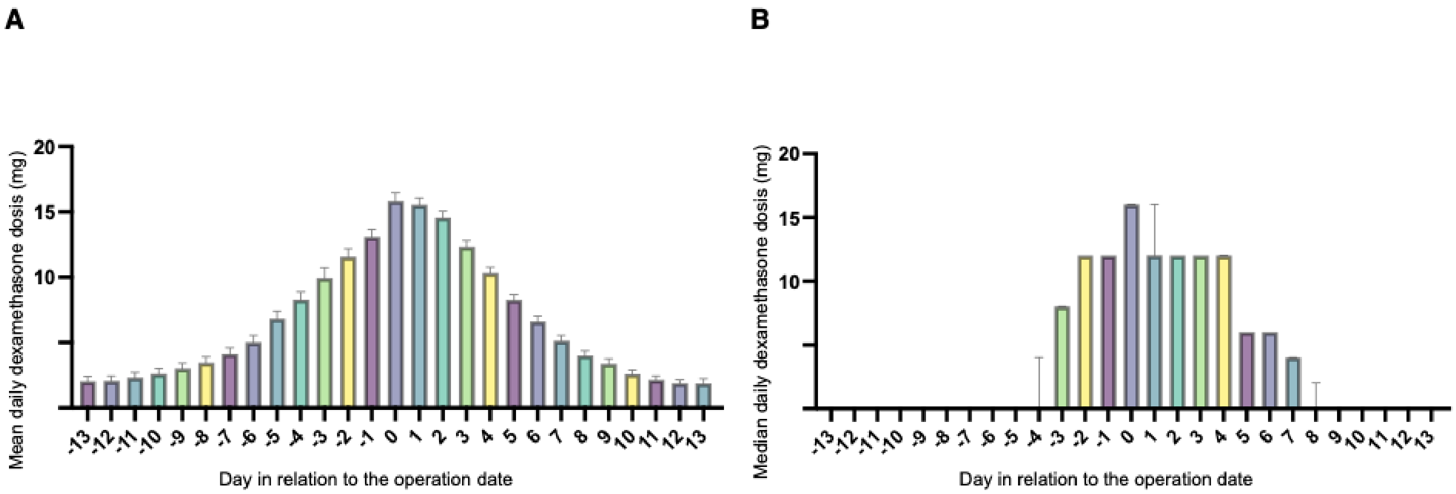

Bar plots for the mean daily peri-operative dexamethasone dosis for all centers (**A**) and the median peri-operative dexamethasone dosis for all centers (**B**); the x axis represent the days before operation (day -13 – day -1), the operation data, which is day 0, and the days after operation (day 1 – day 13).

© 2025 Wasilewski D et al. *JAMA Network Open*.

eFigure 6. Time Course of Mean Perioperative Dexamethasone per Center

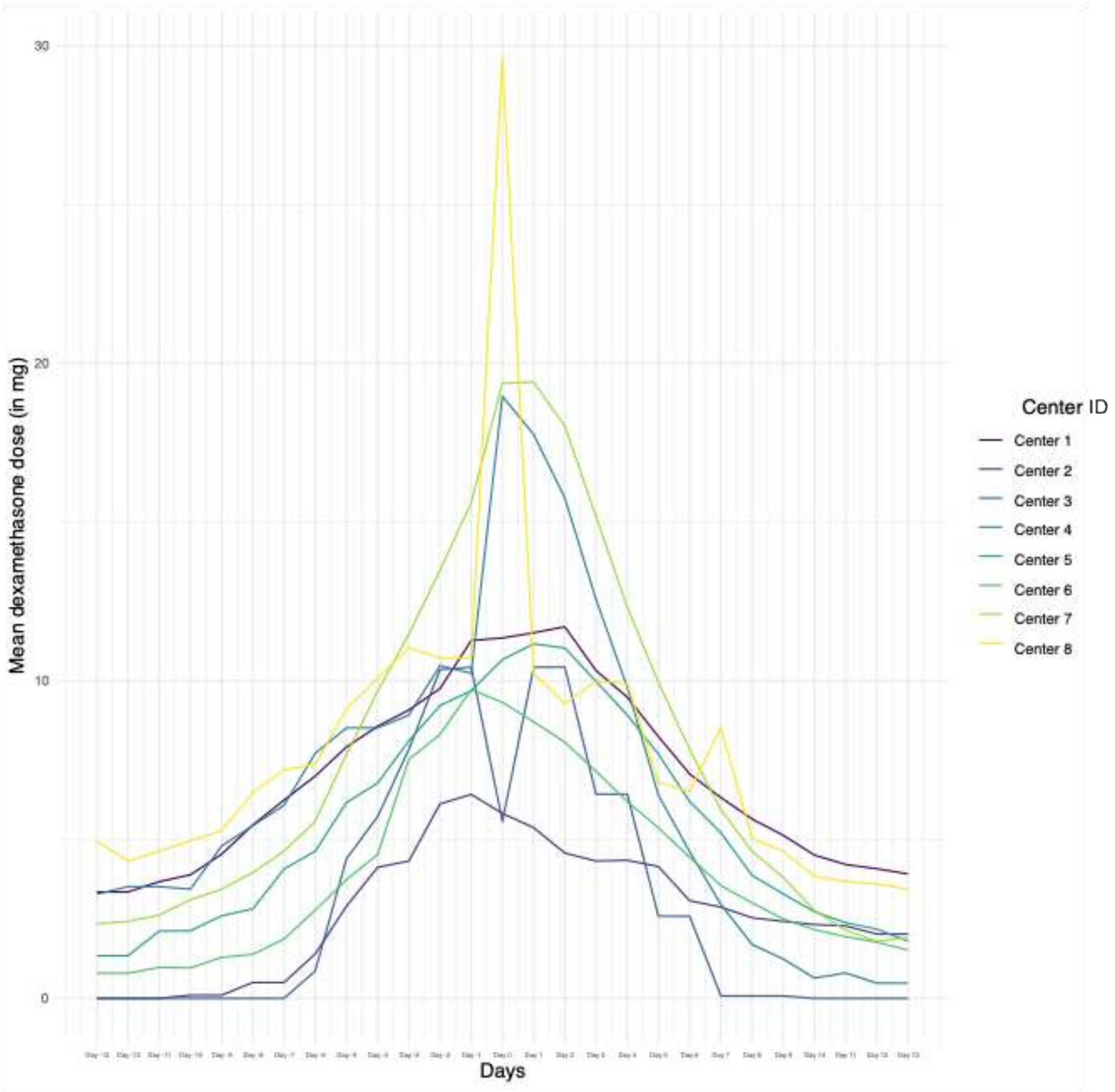

© 2025 Wasilewski D et al. *JAMA Network Open*.

Line plots depicting the time course of mean per-operative dexamethasone (in mg) for each of the participating centers; the x axis represents the days before operation (day -13 – day -1), the operation data which is day 0, and the days after operation (day 1 – day 13).

© 2025 Wasilewski D et al. *JAMA Network Open*.

eFigure 7. Correlations Between Numerical Clinical Covariates

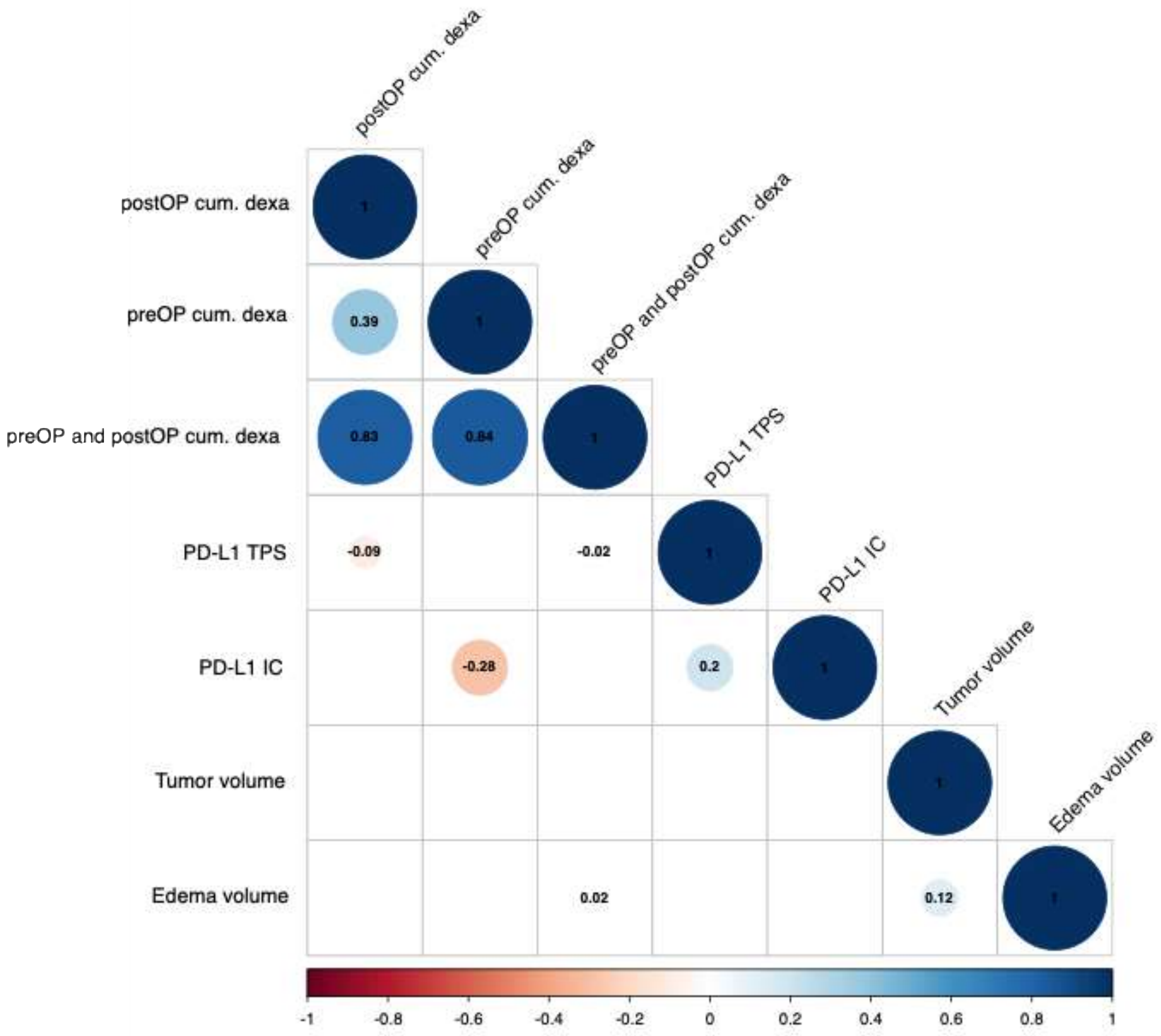

A correlation plot visualizes Pearson correlation coefficients between clinical covariates, including pre-operative cumulative dexamethasone (preOP.cum.dexa), post-operative cumulative dexamethasone (postOP cum.dexa), peri-operative cumulative

© 2025 Wasilewski D et al. *JAMA Network Open*.

dexamethasone (preOP and postOP cum-dexa), Brain metastasis-specific programmed death ligand 1 (PD-L1) tumor proportion score (TPS) (PD-L1 TPS), Brain metastasis-specific programmed death ligand 1 (PD-L1) immune cell (IC) score (PD-L1 IC), tumor volume (Tumor volume), and edema volume (Edema volume). Key correlations include a strong positive relationship between pre-operative and post-operative cumulative dexamethasone doses (r = 0.84), indicating a consistent dexamethasone administration pattern surrounding surgical procedures. A notable positive correlation is also observed between pre-operative cumulative dexamethasone dose and peri-operative cumulative dexamethasone dose (r = 0.84), underscoring the role of pre-operative treatment in overall peri-operative management. Conversely, correlations between PD-L1 scores (both TPS and IC) and dexamethasone doses are weaker, with coefficients ranging from -0.28 to 0.20, suggesting a less direct relationship between immune marker expression and steroid use. Tumor and edema volumes show minimal to no correlation with dexamethasone doses, with coefficients close to zero, indicating that tumor size and associated edema are not strongly related to the cumulative dexamethasone dose in this dataset.

© 2025 Wasilewski D et al. *JAMA Network Open*.

eFigure 8. Association Between Cumulative Dexamethasone and Postoperative Wound Revision Surgery

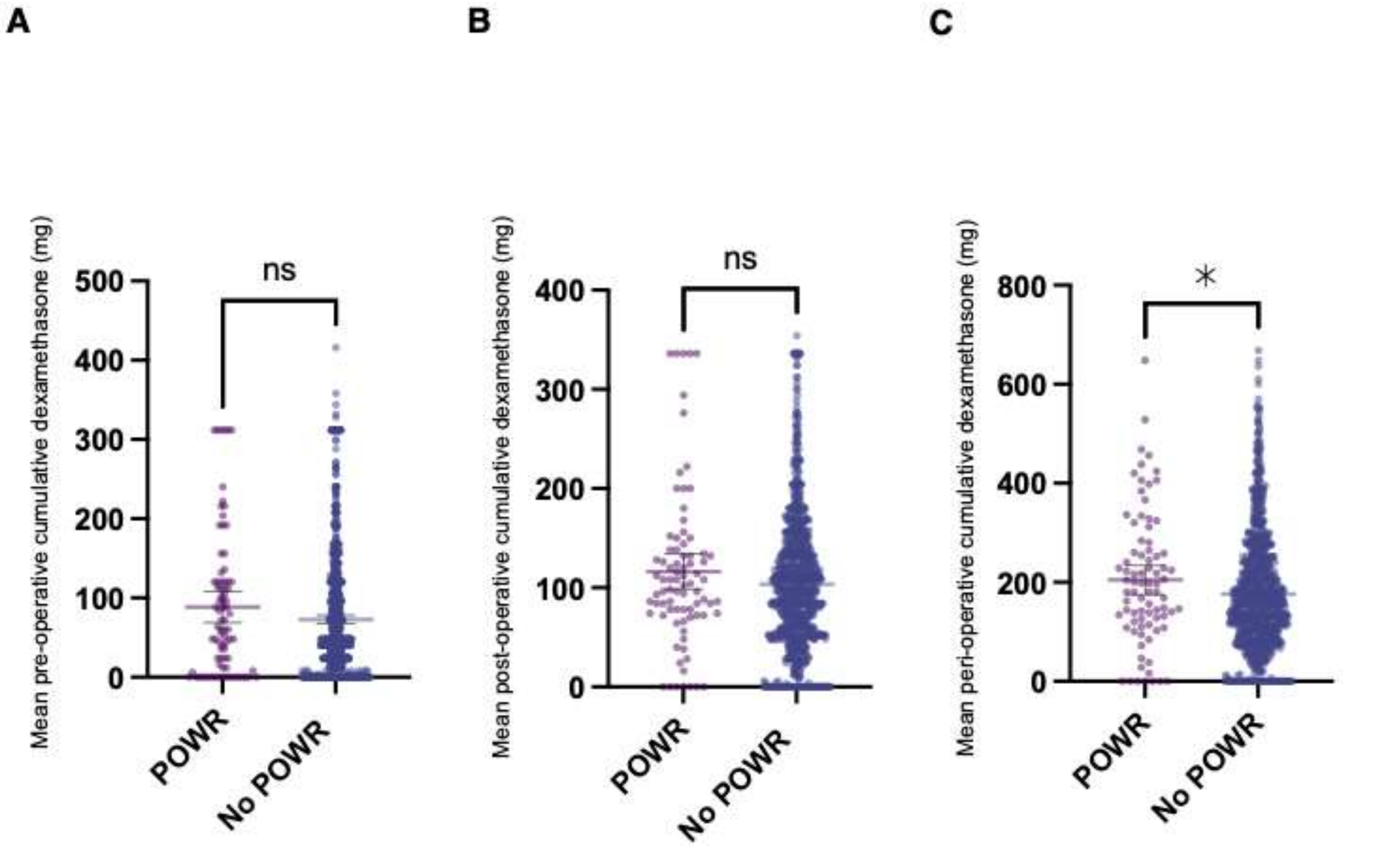

Bar graphs comparing the mean cumulative doses of peri-operative (**A**), pre-operative (**B**), and post-operative (**C**) dexamethasone (in milligrams) for patients requiring post-operative wound revision surgery (POWR) (any wound revision surgery) or no POWR. Differences were compared using two-tailed Mann-Whitney test; results were considered statistically significant for p values <0.05.

© 2025 Wasilewski D et al. *JAMA Network Open*.

eFigure 9. Cut Point Selection Using Maximally Selected Rank Statistics

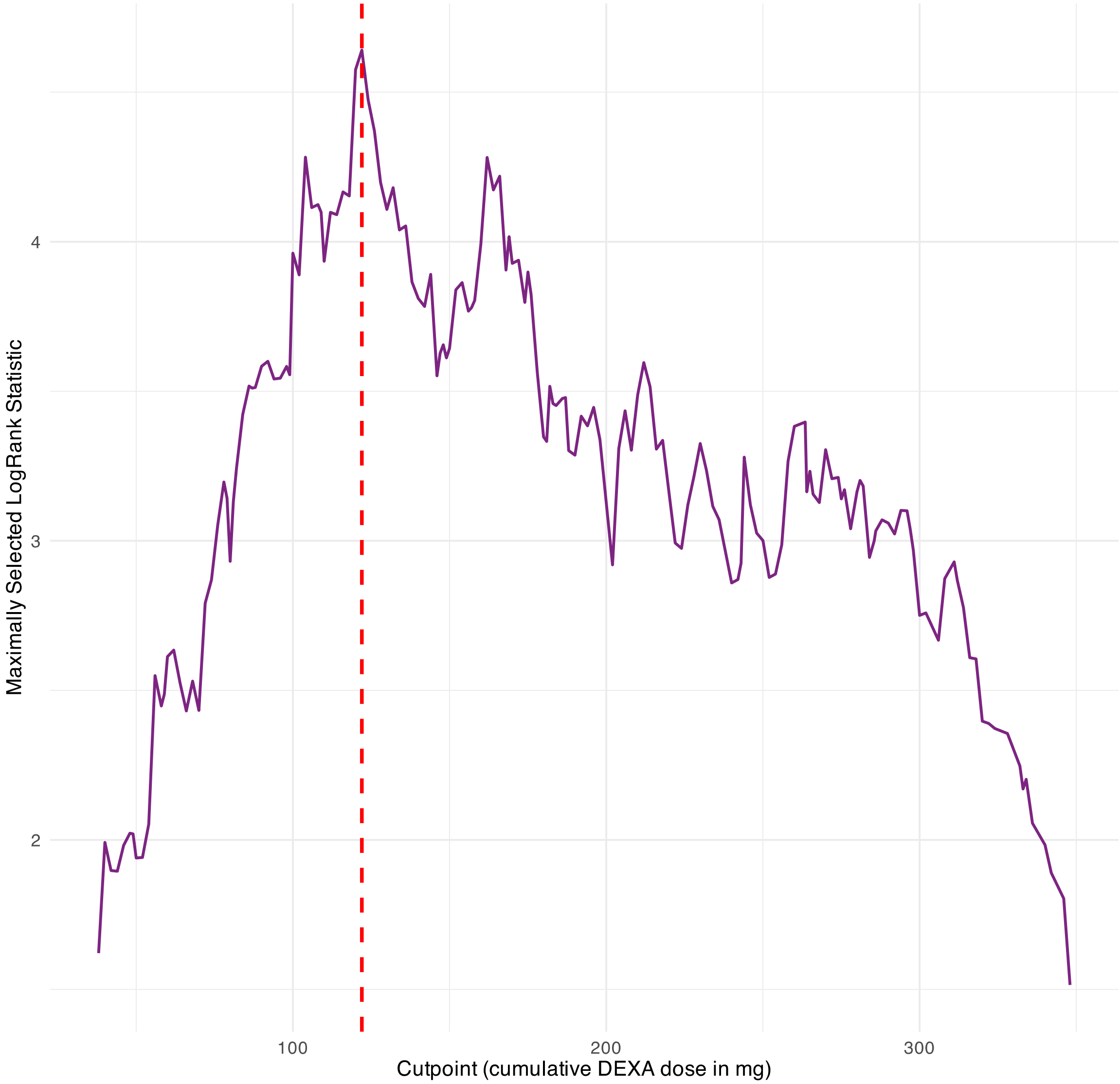

Cutpoint selection for the predictor variable cumulative peri-operative dexamethasone on OS as an outcome parameter using maximally selected rank statistics in the total cohort (n=1064) identified a cutpoint of 122 mg (Maximally selected rank

© 2025 Wasilewski D et al. *JAMA Network Open*.

statistics=4.6408, p=7.545e-05). Cutpoints for cumulative peri-operative dexamethasone for ecPFS was 166 mg (Maximally selected rank statistics=3.486, p=0.009083), and 104 mg for icPFS (Maximally selected rank statistics=4.014, p=0.001607). Optimal cutpoint for cumulative pre-operative dexamethasone for OS was 48 mg (Maximally selected rank statistics=3.7242, p=0.002911), for ecPFS was 96 mg (Maximally selected rank statistics=3.1638, p=0.01793), and for icPFS the optimal cutpoint was 104 mg (Maximally selected rank statistics=3.2792, p=0.01243). Optimal cutpoint for post-operative cumulative dexamethasone for OS was 56 mg (Maximally selected rank statistics=3.6362, p=0.004978), for ecPFS was 92 mg (Maximally selected rank statistics=2.3701, p=0.1837), and for icPFS was 92 mg (Maximally selected rank statistics=3.0971, p=0.02982) (data not shown).

© 2025 Wasilewski D et al. *JAMA Network Open*.

eFigure 10. Quartiles and Spline Regression to Model Dose-Dependency of Cumulative Perioperative Dexamethasone on the Hazard of Death

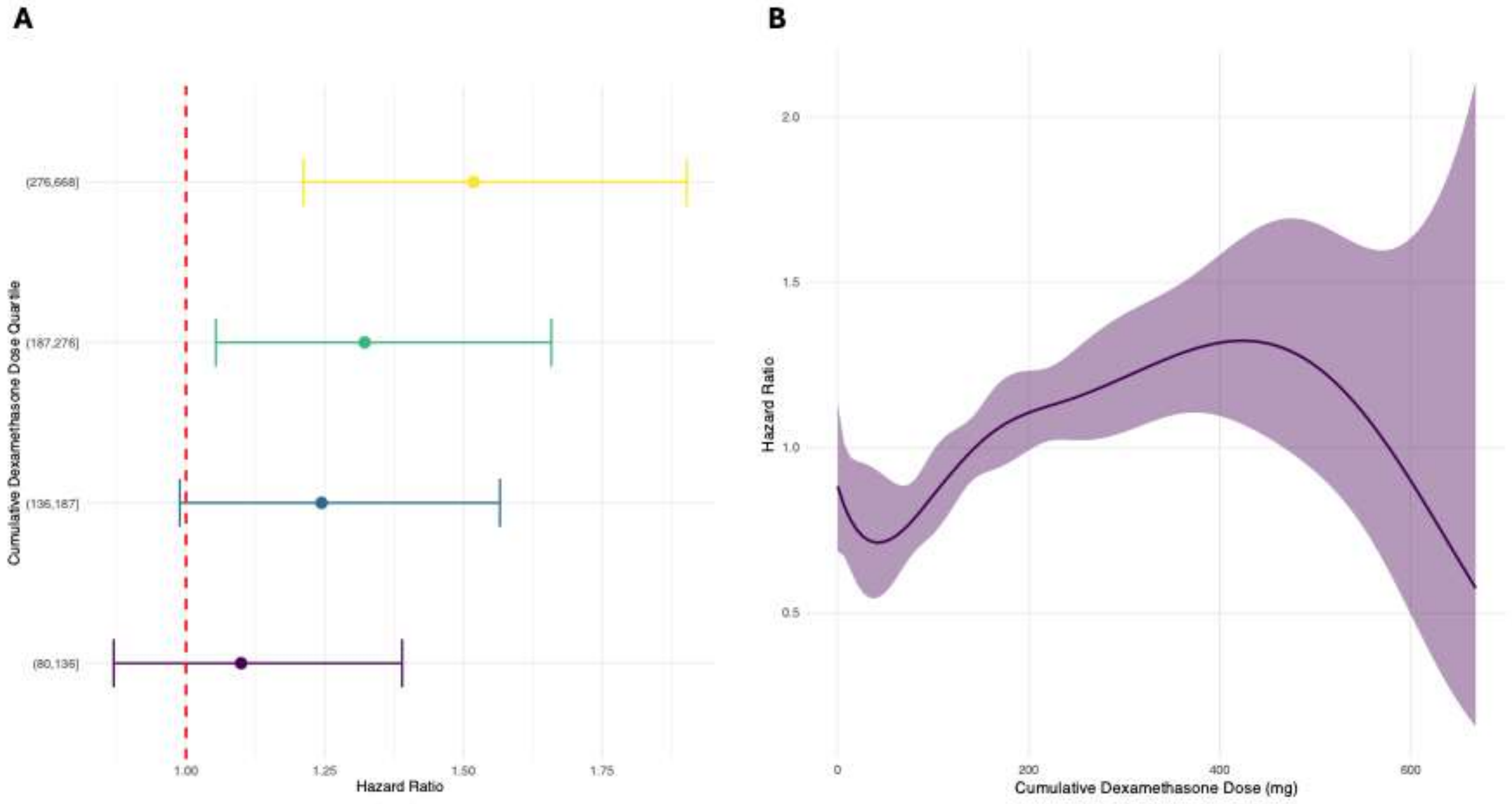

Figure A shows relationship of Hazard ratios and quartiles of cumulative dexamethasone (**A**), whereas Figure B illustrates the estimated hazard ratio (HR) for survival as a function of cumulative dexamethasone dose (mg), derived from a Cox proportional hazards model with cubic spline terms (**B**). The model was fitted using cumulative dexamethasone dose as the predictor,

© 2025 Wasilewski D et al. *JAMA Network Open*.

incorporating spline knots at the 25th, 50th, and 75th percentiles to accommodate flexibility in the dose-response relationship. The solid violet line represents the spline-estimated hazard ratio across varying doses, while the shaded area signifies the 95% confidence interval for the HR estimate, highlighting the associated uncertainty. The analysis utilized a dataset with N=1064 observations. The likelihood ratio test for the model's overall significance yielded a p-value of 0.0007, indicating a significant association between cumulative dexamethasone dose and survival when accounting for non-linear effects. Model fit was assessed using the Akaike Information Criterion (AIC) and the Bayesian Information Criterion (BIC), which were 9851.756 and 9879.826, respectively, demonstrating a satisfactory fit to the observed data. The concordance index was 0.567 (se=0.011), supporting the model's predictive capability.

© 2025 Wasilewski D et al. *JAMA Network Open*.

eFigure 11. Tumor-Specific Subgroup KM Analysis for Dexamethasone 122-mg Cutoff—Unmatched Data

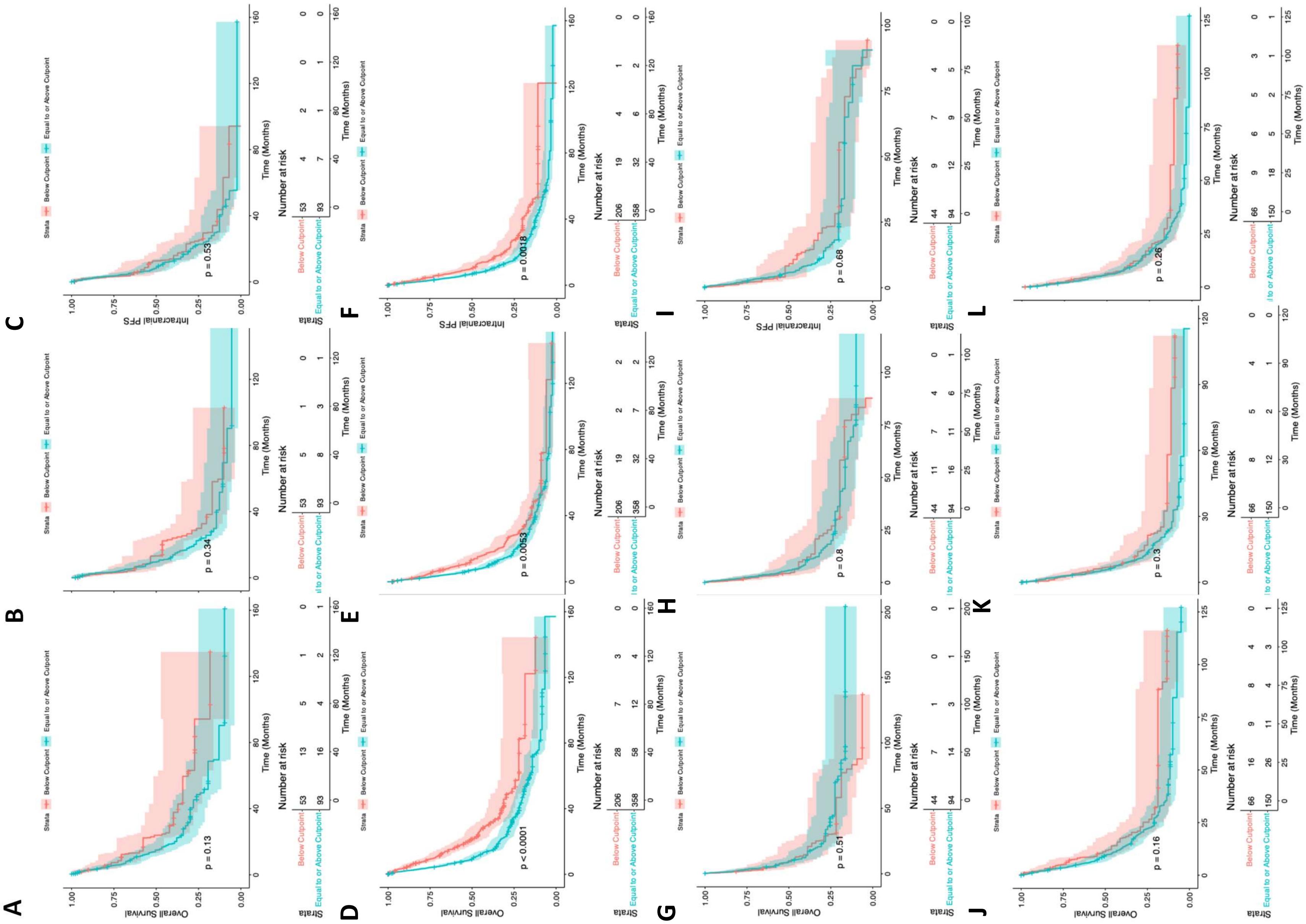

© 2025 Wasilewski D et al. *JAMA Network Open*.

Panels a-c: Subgroup analysis for breast cancer demonstrates Kaplan-Meier survival curves and forest plots. Panel a illustrates the overall survival (OS) curves comparing "Below Cutpoint" and "Equal to or Above Cutpoint" dexamethasone groups, showing a median OS of 23.9 months and 14.9 months, respectively. Panel b displays extracranial progression-free survival (ecPFS), with median survival of 13.2 months for the "Below Cutpoint" group versus 8.47 months for the "Equal to or Above Cutpoint" group. Panel c highlights intracranial progression-free survival (icPFS), revealing medians of 12.6 months and 7.63 months for the respective groups.

Panels d-f: Subgroup analysis for NSCLC. Panel d depicts OS, with the "Below Cutpoint" group having a significantly longer median survival of 21.7 months compared to 12.5 months in the "Equal to or Above Cutpoint" group ($p < 0.01$). Panel e shows ecPFS, with median survivals of 13.27 months and 7.93 months, respectively, favoring the "Below Cutpoint" group ($p < 0.05$). Panel f illustrates icPFS, with medians of 10.37 months and 7.87 months for the two groups, with no significant difference.

Panels g-i: Subgroup analysis for melanoma. Panel g shows OS, where both groups demonstrate overlapping curves with median survivals of 9.17 months ("Below Cutpoint") and 10.87 months ("Equal to or Above Cutpoint"). Panel h highlights ecPFS, showing similar trends with no significant differences. Panel i illustrates icPFS, where survival medians are 8.30 months and 6.07 months for the respective groups.

Panels j-l: Subgroup analysis for "Other" cancer types. Panel j presents OS, with medians of 11.10 months ("Below Cutpoint") and 7.57 months ("Equal to or Above Cutpoint"). Panel k shows ecPFS, where both groups overlap significantly. Panel l illustrates icPFS, with medians of 6.80 months and 5.27 months for the two groups.

© 2025 Wasilewski D et al. *JAMA Network Open*.

eFigure 12. Tumor-Specific Subgroup Cox Proportional Hazard Regression Analysis for Dexamethasone 122-mg Cutoff—Unmatched Data

A

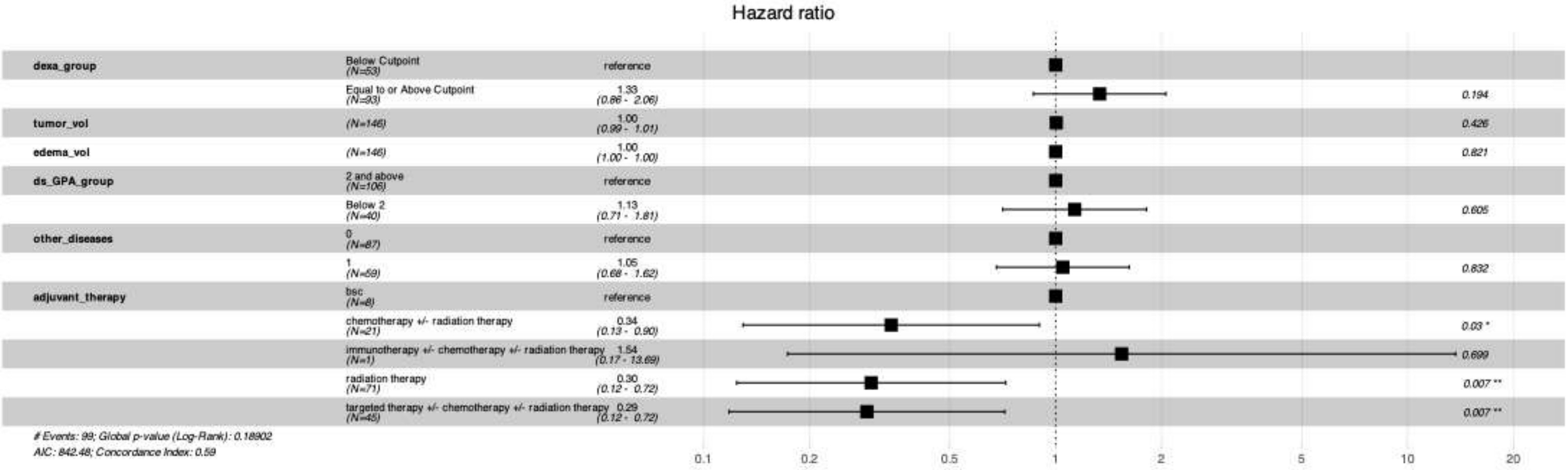

B

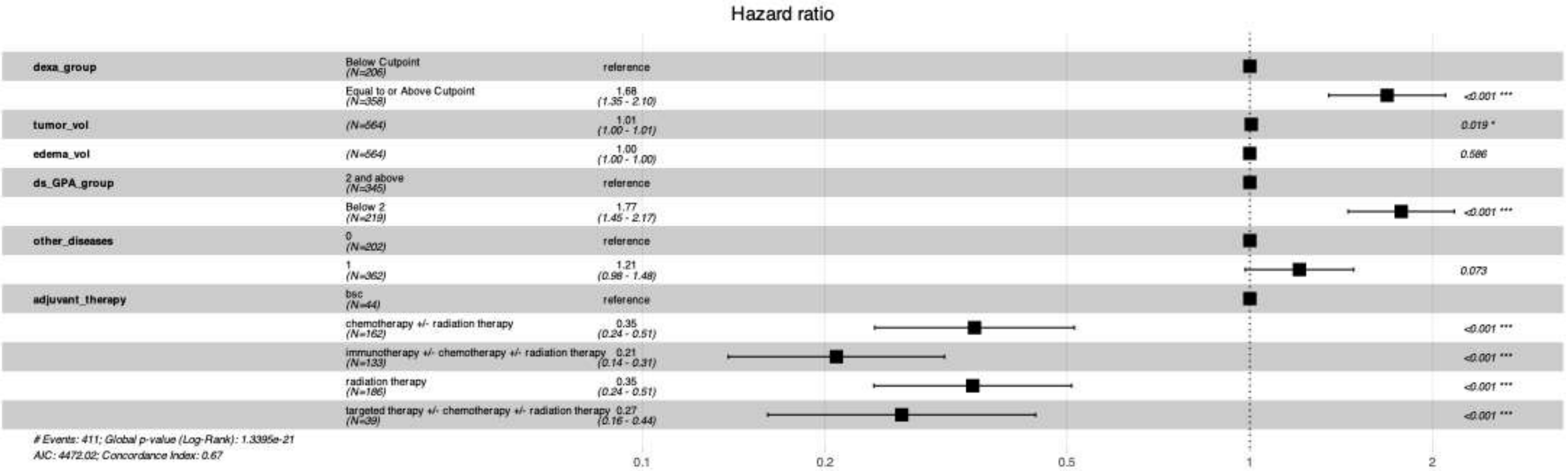

© 2025 Wasilewski D et al. *JAMA Network Open*.

C

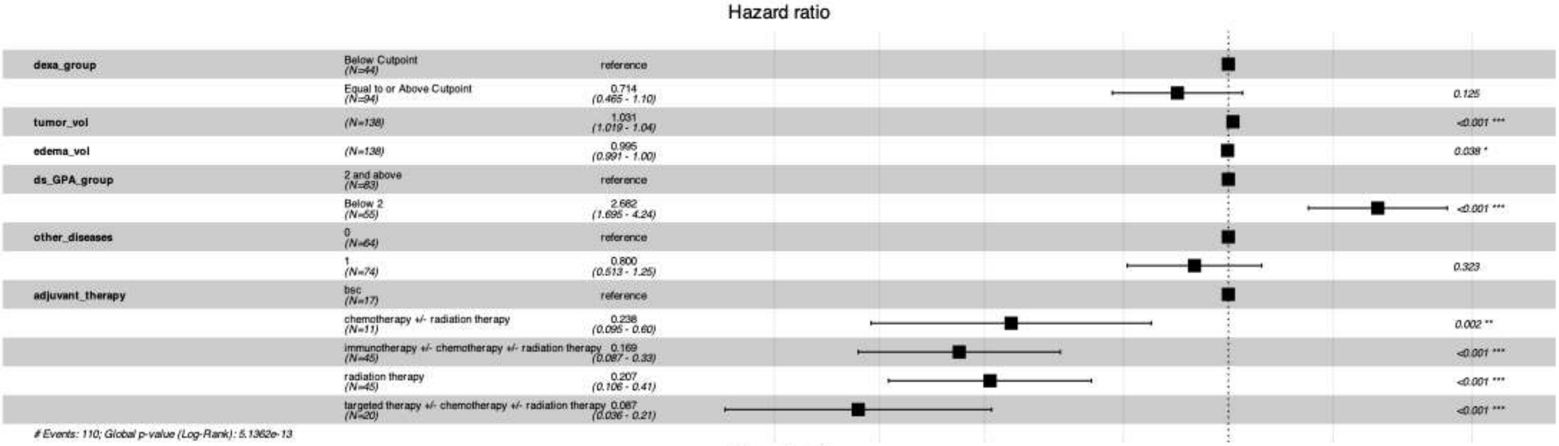

D

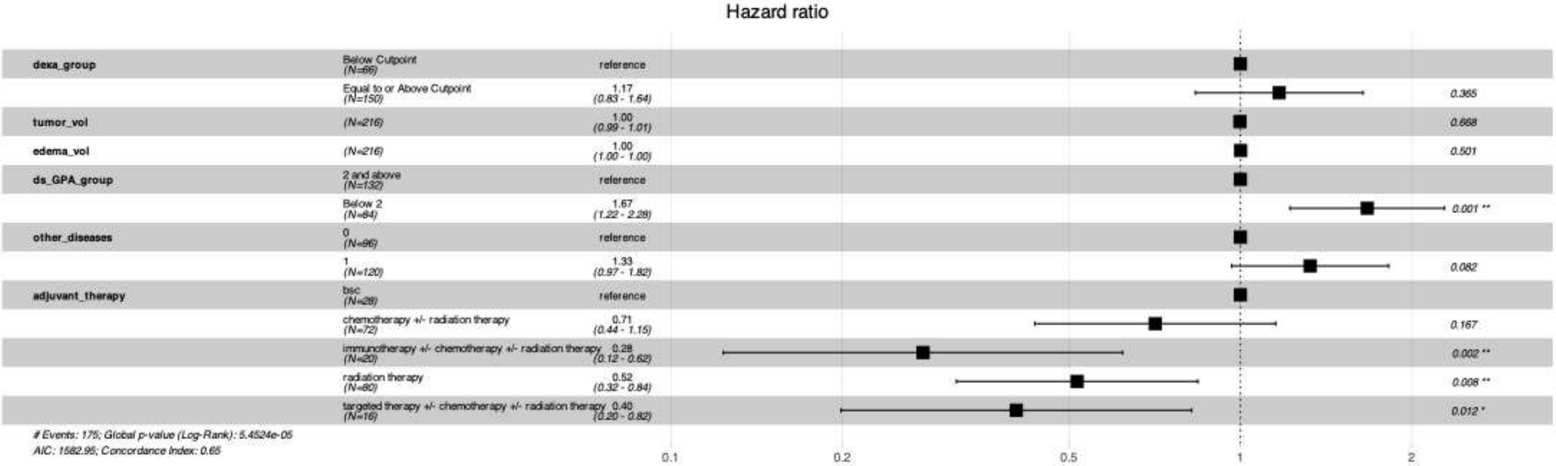

Panels a-d: Panel A shows the Cox proportional hazards model for OS unmatched data for the subgroup of breast cancer patients (A), panel B for NSCLC (B), panel C for melanoma (C) and panel D for other cancer entities (D). In case of NSCLC the selected cutpoint dichotomizing patients into low and high peri-operative dexamethasone was significantly associated with OS, whereas in breast cancer patients, melanoma patients and patients with other entities this association was lacking. ds-GPA was porgnostic in all entities apart from breatst cancer. Post-operative treatment, especially that including targeted treatment or immunotherapy showed the strongest survival benefit across all cancer entities.

© 2025 Wasilewski D et al. *JAMA Network Open*.

eFigure 13. Tumor-specific Subgroup KM Analysis for Dexamethasone 122-mg Cutoff—Matched Data

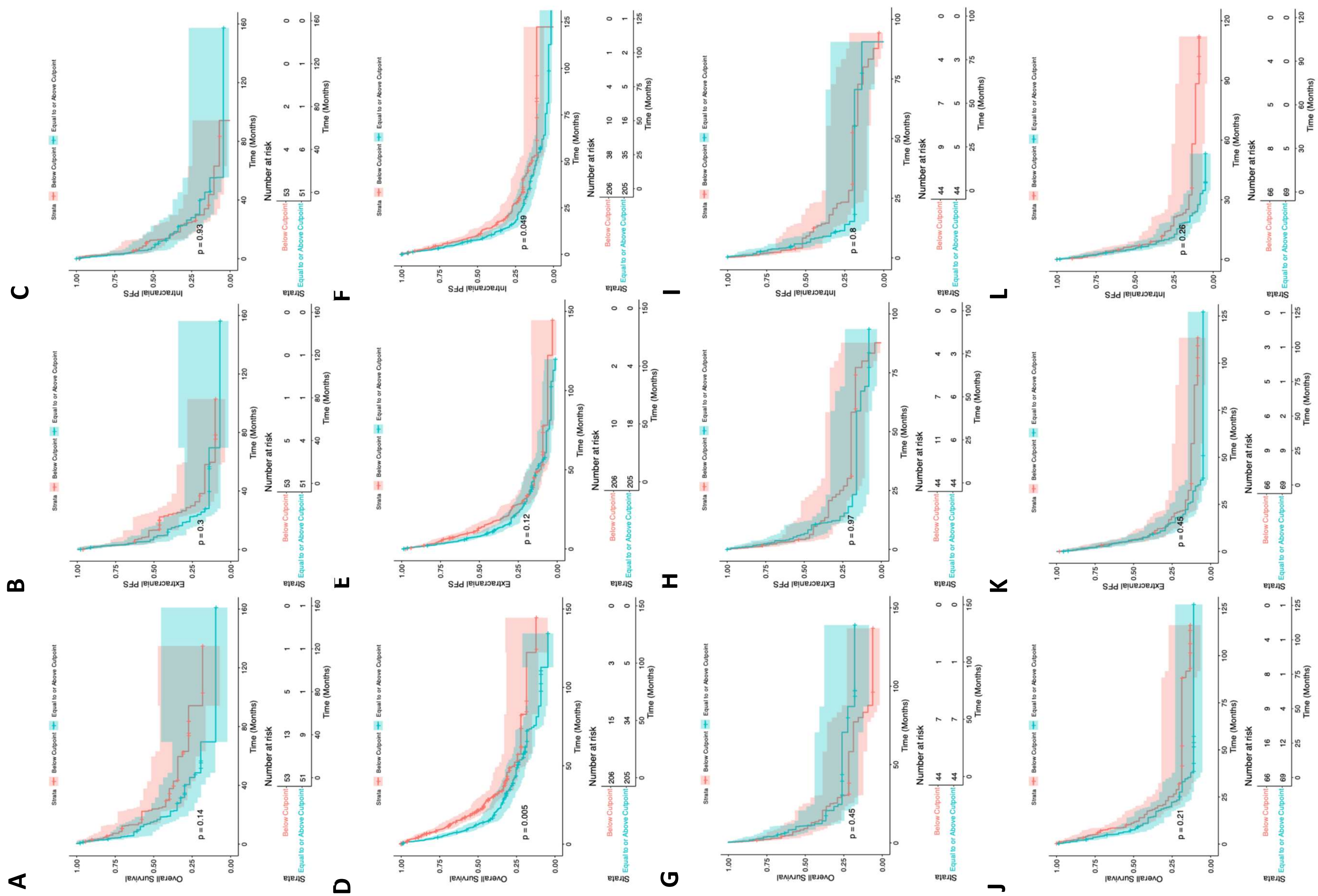

© 2025 Wasilewski D et al. *JAMA Network Open*.

Panels a-c: Subgroup analysis for breast cancer after matching demonstrates consistent trends with unmatched data. Panel a shows OS, where the "Below Cutpoint" group retains a longer median OS (23.9 months) compared to the "Equal to or Above Cutpoint" group (14.9 months). Panel b illustrates ecPFS, with medians of 13.2 months and 8.47 months for the two groups. Panel c shows icPFS, with medians of 12.6 months and 7.63 months, respectively.

Panels d-f: Subgroup analysis for NSCLC after matching. Panel d shows OS, with a significant difference favoring the "Below Cutpoint" group (21.7 months vs. 12.5 months; $p < 0.01$). Panel e highlights ecPFS, with medians of 13.27 months and 7.93 months for the two groups ($p < 0.05$). Panel f illustrates icPFS, showing consistent trends with medians of 10.37 months and 7.87 months.

Panels g-i: Subgroup analysis for melanoma in matched data. Panel g depicts OS with overlapping survival curves. Panel h shows ecPFS, where no significant differences are observed. Panel i illustrates icPFS, showing consistent survival trends across groups.

Panels j-l: Subgroup analysis for "Other" cancer types in matched data. Panel j shows OS, with medians of 11.10 months ("Below Cutpoint") and 7.57 months ("Equal to or Above Cutpoint"). Panel k depicts ecPFS, with overlapping curves. Panel l illustrates icPFS, showing similar survival medians

© 2025 Wasilewski D et al. *JAMA Network Open*.

eFigure 14. Tumor-Specific Subgroup Cox Proportional Hazard Regression Analysis for Dexamethasone 122-mg Cutoff—Matched Data

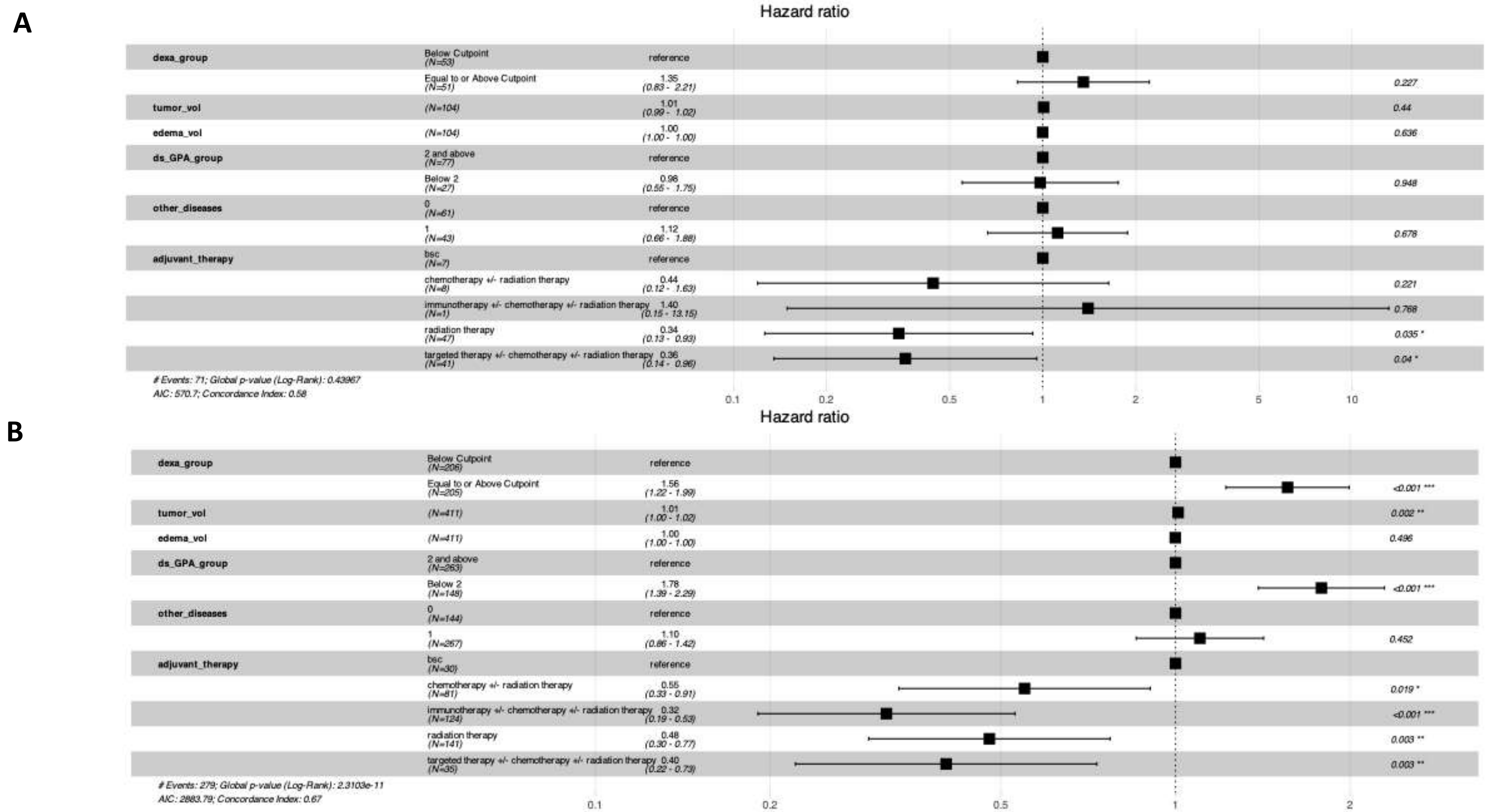

© 2025 Wasilewski D et al. *JAMA Network Open*.

C

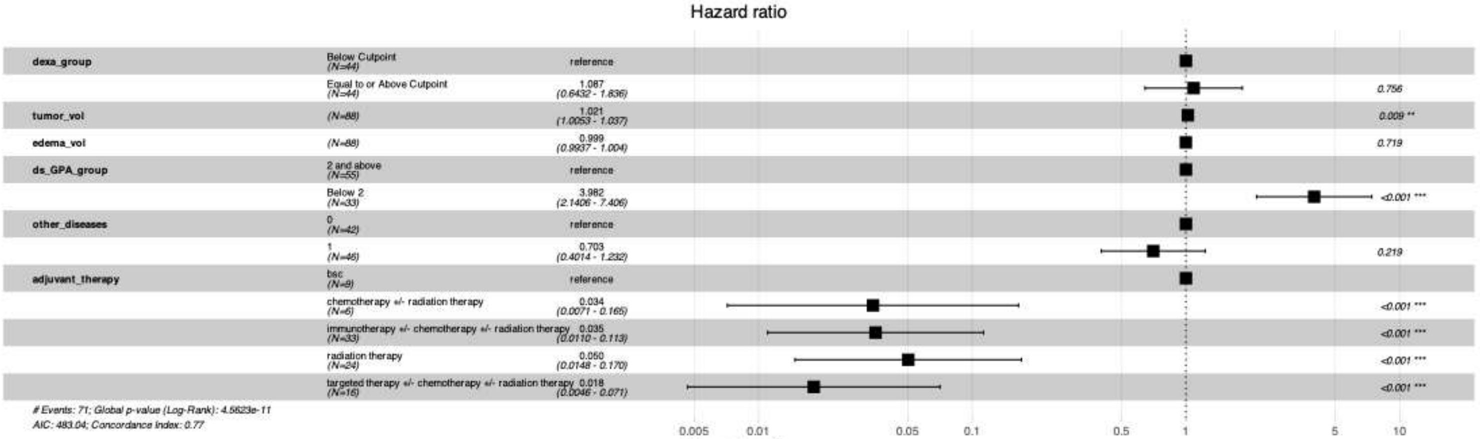

D

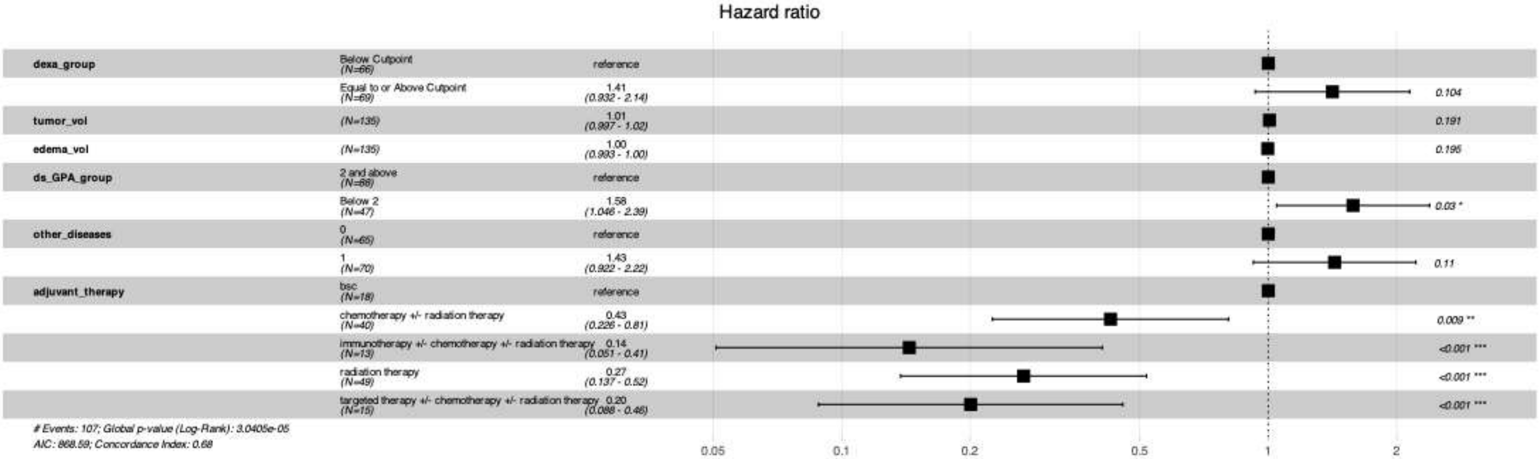

Panels a-d: Panel A shows the Cox proportional hazards model for OS matched data for the subgroup of breast cancer patients (A), panel B for NSCLC (B), panel C for melanoma (C) and panel D for other cancer entities (D). Similar to unmatched data in the case of NSCLC the selected cutpoint dichotomizing patients into low and high peri-operative dexamethasone was significantly associated with OS, whereas in breast cancer patients, melanoma patients and patients with other entities this association was lacking.

© 2025 Wasilewski D et al. *JAMA Network Open*.

eTable 1. Patient Characteristics of the Total Cohort Before Matching

**Characteristic**	**Overall** N = 1,064	**Below Cutpoint** N = 369	**Equal to or Above Cutpoint** N = 695	**p-value**[1]	**q-value**[2]
**Age, Median (IQR)**	64 (56 – 72)	63 (55 – 72)	65 (56 – 72)	0.29	0.37
**Gender, n (%)**				0.39	0.44
Female	523 (49)	188 (51)	335 (48)		
Male	541 (51)	181 (49)	360 (52)		
**KPS group, n (%)**				0.001	0.004
≥ 70	871 (83)	322 (88)	549 (80)		
< 70	181 (17)	44 (12)	137 (20)		
**ds-GPA group, n (%)**				0.014	0.031
≥ 2	569 (63)	186 (69)	383 (60)		
< 2	340 (37)	85 (31)	255 (40)		
**Pre-operative cumulative dexamethasone, Median (IQR)**	48 (12 – 112)	8 (0 – 32)	88 (48 – 144)	<0.001	<0.001
**Post-operative cumulative dexamethasone, Median (IQR)**	98 (56 – 137)	48 (12 – 70)	124 (96 – 160)	<0.001	<0.001
**Peri-operative cumulative dexamethasone, Median (IQR)**	158 (96 – 240)	72 (24 – 99)	208 (160 – 288)	<0.001	<0.001

© 2025 Wasilewski D et al. *JAMA Network Open*.

Characteristic	Overall<br>N = 1,064	Below Cutpoint<br>N = 369	Equal to or Above Cutpoint<br>N = 695	p-value[1]	q-value[2]
**Primary tumor resection, n (%)**				0.18	0.24
No primary tumor resection	399 (50)	123 (49)	276 (50)		
Before brain metastasis resection	355 (44)	120 (47)	235 (43)		
After brain metastasis resection	48 (6.0)	10 (4.0)	38 (6.9)		
**Adjuvant treatment after first brain metastasis resection, n (%)**				0.002	0.007
Best supportive care	97 (9.2)	32 (8.8)	65 (9.4)		
Chemotherapy +/- radiation therapy	264 (25)	72 (20)	192 (28)		
Immunotherapy +/- chemotherapy +/- radiation therapy	197 (19)	81 (22)	116 (17)		
Radiation therapy	378 (36)	126 (35)	252 (37)		
Targeted therapy +/- chemotherapy +/- radiation therapy	119 (11)	54 (15)	65 (9.4)		
**Tumor volume, Median (IQR)**	12 (6 – 23)	11 (6 – 22)	13 (7 – 23)	0.11	0.19
**Edema volume, Median (IQR)**	54 (26 – 98)	47 (20 – 85)	57 (28 – 104)	0.004	0.011
**Number of brain metastases, n (%)**				0.016	0.031
1	493 (47)	193 (53)	300 (44)		
2	273 (26)	88 (24)	185 (27)		

© 2025 Wasilewski D et al. *JAMA Network Open*.

**Characteristic**	**Overall**<br>N = 1,064	**Below Cutpoint**<br>N = 369	**Equal to or Above Cutpoint**<br>N = 695	**p-value**[1]	**q-value**[2]
≥ 2	284 (27)	84 (23)	200 (29)		
**Brain metastasis location, n (%)**				0.15	0.22
Supratentorial	647 (61)	222 (61)	425 (61)		
Infratentorial	198 (19)	78 (21)	120 (17)		
Both	212 (20)	64 (18)	148 (21)		
**Presence of extracranial metastases at time of brain metastasis resection, n (%)**	539 (52)	190 (53)	349 (51)	0.57	0.61
**Presence of other non-oncological diseases, n (%)**	564 (58)	204 (59)	360 (58)	0.74	0.74
**Entity, n (%)**					
Breast cancer	146 (14)	53 (14)	93 (13)		
Cervical cancer	4 (0.4)	0 (0)	4 (0.6)		
Cholangiocarcinoma	1 (<0.1)	0 (0)	1 (0.1)		
Colorectal cancer	34 (3.2)	8 (2.2)	26 (3.7)		
CUP	8 (0.8)	2 (0.5)	6 (0.9)		
Endometrial cancer	3 (0.3)	3 (0.8)	0 (0)		
Esophageal cancer	5 (0.5)	2 (0.5)	3 (0.4)		
Gastric cancer	2 (0.2)	0 (0)	2 (0.3)		
Head and neck cancer	7 (0.7)	4 (1.1)	3 (0.4)		

© 2025 Wasilewski D et al. *JAMA Network Open*.

Characteristic	Overall<br>N = 1,064	Below Cutpoint<br>N = 369	Equal to or Above Cutpoint<br>N = 695	p-value[1]	q-value[2]
Leiomyosarcoma	1 (<0.1)	0 (0)	1 (0.1)		
Melanoma	138 (13)	44 (11.9)	94 (13.6)		
NSCLC	564 (53)	206 (56)	358 (52)		
Esophageal carcinoma	1 (<0.1)	0 (0)	1 (0.1)		
Ovarian cancer	9 (0.8)	4 (1.1)	5 (0.7)		
Pancreatic carcinoma	1 (<0.1)	0 (0)	1 (0.1)		
Pleural mesothelioma	1 (<0.1)	0 (0)	1 (0.1)		
Prostate cancer	3 (0.3)	2 (0.5)	1 (0.1)		
Renal cell cancer	60 (5.7)	18 (4.9)	42 (6.1)		
Sarcoma	2 (0.2)	1 (0.3)	1 (0.1)		
SCLC	64 (5.9)	18 (4.9)	46 (6.6)		
Synovial cancer	1 (<0.1)	1 (0.3)	0 (0)		
Testicular cancer	2 (0.2)	0 (0)	2 (0.3)		
Urothelial cancer	7 (0.7)	3 (0.8)	4 (0.6)		
**Entity group, n (%)**				0.37	0.44
Breast cancer	146 (14)	53 (14)	93 (13)		
Melanoma	138 (13)	44 (12)	94 (14)		
NSCLC	564 (53)	206 (56)	358 (52)		

© 2025 Wasilewski D et al. *JAMA Network Open*.

Characteristic	Overall N = 1,064	Below Cutpoint N = 369	Equal to or Above Cutpoint N = 695	p-value[1]	q-value[2]
Other	216 (20)	66 (18)	150 (22)		
**Surgical site infection, n (%)**				0.073	0.13
No POWR	977 (92)	346 (94)	631 (91)		
POWR	82 (7.7)	21 (5.7)	61 (8.8)		

[1] Wilcoxon rank sum test; Pearson's Chi-squared test

[2] False discovery rate correction for multiple testing

The table presents characteristics of 1064 patients with resected brain metastases, grouped by cumulative peri-operative dexamethasone dosage (< 122 mg vs. ≥ 122 mg), q-value (adjusted p-value) and mean standardized differences of variables considered for PSM before matching are displayed on the right**.** The median age is 64 years with a balanced gender distribution (51% male, 49% female), showing no significant differences in age (p=0.29, q=0.37) or gender (p=0.39, q=0.44) between groups. Patients below the cutpoint had better functional status (88% KPS ≥ 70, p=0.001, q=0.004) and prognosis (69% ds-GPA ≥ 2, p=0.014, q=0.031). Significant differences in dexamethasone dosage were observed: median pre-operative doses were 8 mg (below cutpoint) versus 88 mg (above cutpoint) (p<0.001, q<0.001), with similar trends for cumulative and post-operative doses. The equal to/above cutpoint group more frequently received chemotherapy +/- radiation therapy (28% vs. 20%, p=0.002, q=0.007) and had higher edema volumes (57 mL vs. 47 mL, p=0.004, q=0.011) and more than two brain metastases (29% vs. 23%, p=0.016, q=0.031). The distribution of primary tumor types and surgical site infection rates did not differ significantly between groups (adjusted p value=0.13). Standard mean differences before matching and after matching are displayed in Figure 2A.

© 2025 Wasilewski D et al. *JAMA Network Open*.

Center ID	Number of values	Minimum	25% Percentile	Median	75% Percentile	Maximum	Range	Mean	Std. Deviation	Std. Error of Mean	Lower 95% CI of mean	Upper 95% CI of mean
Center 1	74	0	108	156	271	528	528	187,6351	113,7733	13,22588	161,276	213,9943
Center 2	40	0	40	78	104	160	160	74,55	41,30683	6,531183	61,33944	87,76056
Center 3	51	24	68	76	100	128	104	84,15686	24,59217	3,443593	77,2402	91,07352
Center 4	50	0	113	167	234,5	552	552	178,64	100,6262	14,23069	150,0424	207,2376
Center 5	110	0	84	131	180	600	600	148,1727	99,32625	9,470386	129,4027	166,9427
Center 6	121	0	30	108	162	422	422	110,3967	90,41879	8,21989	94,12188	126,6715
Center 7	593	0	132	190	280	668	668	211,1176	129,2874	5,309197	200,6905	221,5448
Center 8	25	44	168	208	280	428	384	212	96,6954	19,33908	172,0861	251,9139

eTable 2. Statistical Summary of Perioperative Dexamethasone Doses Across Different Centers

© 2025 Wasilewski D et al. *JAMA Network Open*.

Supplementary Table 2 provides a summary of descriptive statistical values for post-operative dexamethasone doses across eight centers. The table includes the number of observations, minimum, 25th percentile, median, 75th percentile, maximum, range, mean, standard deviation, standard error of the mean, and the 95% confidence intervals for the mean. Center 1 (N=74) has a mean dose of 103.53 mg (95% CI: 90.55-116.51) with a range from 0 to 272 mg. Center 2 (N=40) reports a mean dose of 48.05 mg (95% CI: 38.13-57.97) and a range from 0 to 112 mg. Center 3 (N=51) shows a mean dose of 44.71 mg (95% CI: 40.48-48.93) with a range from 12 to 92 mg. Center 4 (N=50) presents a mean dose of 94.16 mg (95% CI: 79.90-108.42) and a range from 0 to 336 mg. Center 5 (N=110) has a mean dose of 87.15 mg (95% CI: 77.94-96.35) with a range from 0 to 336 mg. Center 6 (N=121) reports a mean dose of 65.74 mg (95% CI: 55.82-75.65) and a range from 0 to 252 mg. Center 7 (N=593) shows a mean dose of 125.09 mg (95% CI: 119.13-131.06) with a range from 0 to 354 mg. Center 8 (N=25) has a mean dose of 115.12 mg (95% CI: 93.94-136.30) with a range from 32 to 200 mg. The data illustrate significant variability in post-operative dexamethasone doses across the centers. Center 7, with the most observations, shows the highest mean dose and the widest range. The variability in doses highlights differences in clinical practices or patient needs across the centers. This table is crucial for understanding the distribution and central tendency of post-operative dexamethasone doses in different clinical settings.

© 2025 Wasilewski D et al. *JAMA Network Open*.

eTable 3. Statistical Summary of Preoperative Dexamethasone Doses Across Different Centers

centerID	Number of values	Minimum	25% Percentile	Median	75% Percentile	Maximum	Range	Mean	Std. Deviation	Std. Error of Mean	Lower 95% CI of mean	Upper 95% CI of mean
Center 1	74	0	12	62	133,5	416	416	84,10811	87,21876	10,13897	63,90116	104,3151
Center 2	40	0	7	32	40	64	64	26,5	19,29395	3,050641	20,3295	32,6705
Center 3	51	12	24	40	48	72	60	39,45098	15,69753	2,198094	35,03598	43,86598
Center 4	50	0	6	84	124	312	312	84,48	76,5571	10,82681	62,72271	106,2373
Center 5	110	0	12	48	94	358	358	61,02727	62,43771	5,953202	49,22822	72,82632
Center 6	121	0	0	24	72	312	312	44,66116	51,51012	4,682738	35,38966	53,93265
Center 7	593	0	3,25	54	120	344	344	86,02656	90,55281	3,718558	78,72339	93,32973
Center 8	25	0	44	80	140	264	264	96,88	73,84235	14,76847	66,39937	127,3606

© 2025 Wasilewski D et al. *JAMA Network Open*.

Supplementary Table 3 presents statistical data on pre-operative cumulative dexamethasone from seven different centers. It includes the count of values, as well as the minimum, 25th percentile, median, 75th percentile, maximum, range, mean, standard deviation, standard error of mean, and the 95% confidence intervals for the mean. Center 7 has the most observations (593), while Center 8 has the fewest (40). The data reflects variability in the pre-operative dexamethasone dosing distribution, with Center 1 recording the highest maximum value (416mg) and Center 3 the lowest (72 mg). This table effectively illustrates the spread and central tendencies of the variable across the centers.

© 2025 Wasilewski D et al. *JAMA Network Open*.

eTable 4. Statistical Summary of Postoperative Dexamethasone Doses Across Different Centers

center ID	Number of values	Minimum	25% Percentile	Median	75% Percentile	Maximum	Range	Mean	Std. Deviation	Std. Error of Mean	Lower 95% CI of mean	Upper 95% CI of mean
Center 1	74	0	64	105	135,5	272	272	103,527	56,02109	6,512319	90,54799	116,5061
Center 2	40	0	23	47	62,5	112	112	48,05	31,00533	4,902374	38,13401	57,96599
Center 3	51	12	28	52	52	92	80	44,70588	15,02437	2,103833	40,48021	48,93156
Center 4	50	0	65	96	113,5	336	336	94,16	50,18145	7,096729	79,89859	108,4214
Center 5	110	0	60	75	113,5	336	336	87,14545	48,72349	4,645603	77,93802	96,35289
Center 6	121	0	22	60	96	252	252	65,73554	55,07189	5,006535	55,82295	75,64813
Center 7	593	0	84	114	156	354	354	125,0911	73,96471	3,037367	119,1257	131,0564
Center 8	25	32	76	112	152	200	168	115,12	51,30003	10,26001	93,94439	136,2956

© 2025 Wasilewski D et al. *JAMA Network Open*.

Supplementary Table 4 provides a summary of statistical values for post-operative measure across seven different centers. The table indicates the number of observations, the minimum, 25th percentile, median, 75th percentile, maximum, range, mean, standard deviation, standard error of mean, and the 95% confidence intervals for the mean. The data set is consistent across centers in terms of the number of values, with Center 7 having the most data points. The range of the measure spans from 0 to 354 mg at Center 7, demonstrating significant variability in the data across all centers. The table is instrumental for understanding the distribution and central tendency of the measured variable within each center

© 2025 Wasilewski D et al. *JAMA Network Open*.

eTable 5. Summary of Cut Point Values of Perioperative, Preoperative, and Postoperative Dexamethasone for Patient Outcome Using Maximally Selected Rank Statistics

	Maximally selected rank statistics	p value	Optimal cutpoint (cumulative dexamethasone (in mg))
**Peri-operative cumulative dexamethasone**			
Median OS	4.6408	0.0001558	122
Median ecPFS	3.486	0.009317	166
Median icPFS	4.014	0.002	104
**Pre-operative cumulative dexamethasone**			
Median OS	3.7242	0.002879	48
Median ecPFS	3.1638	0.01778	96
Median icPFS	3.2792	0.01329	104
**Post-operative cumulative dexamethasone**			
Median OS	3.6362	0.005298	56
Median ecPFS	2.3701	0.185	92

© 2025 Wasilewski D et al. *JAMA Network Open*.

Median icPFS	3.0971	0.02915	92

Supplementary Table 5 summarizes the maximally selected rank statistics for peri-operative, pre-operative, and post-operative cumulative dexamethasone, relating them to median OS, extracranial progression-free survival ecPFS, and icPFS. It demonstrates statistically significant association between peri-operative dexamethasone and median OS with a p-value of 0.002024.

© 2025 Wasilewski D et al. *JAMA Network Open*.
