# Supplementary material for: Practice Variation in Perioperative Dexamethasone Use and Outcomes in Brain Metastasis Resection
Source: JAMA Netw Open. 2025 Apr 11;8(4):e254689. doi: 10.1001/jamanetworkopen.2025.4689 (PMC11992604; doi:10.1001/jamanetworkopen.2025.4689)
Supplement: Supplement 2. — Data Sharing Statement [file jamanetwopen-e254689-s002.pdf]

Data Sharing Statement

Wasilewski. Practice Variation in Perioperative Dexamethasone Use and Outcomes in Brain Metastasis Resection. *JAMA Netw Open*. Published April 11, 2025.
doi:10.1001/jamanetworkopen.2025.4689

Data
Data available: Yes
Data types: Deidentified participant data
How to access data: The R code and the raw (deidentified data) will be made available on a repository (github: https://github.com/dasilew/clinicalNSCLC_1)
When available: With publication

Supporting Documents
Document types: Statistical/analytic code
How to access documents: The R code and the raw (deidentified data) will be made available on a repository (github: https://github.com/dasilew/clinicalNSCLC_1)
When available: With publication

Additional Information
Who can access the data: Data will be made available to researchers in the field upon reasonable request via E-Mail.
Types of analyses: R code will include: descriptive statistical data analysis, clinical table creation, Kaplan-Meier analyses, PSM, Cox proportional hazard modelling.
Mechanisms of data availability: Data will be made available with a signed access agreement.
